# The Effect of Drought on Transcriptome and Hormonal Profiles in Barley Genotypes With Contrasting Drought Tolerance

**DOI:** 10.3389/fpls.2020.618491

**Published:** 2020-12-23

**Authors:** Amal Harb, Craig Simpson, Wenbin Guo, Ganesan Govindan, Vijaya Gopal Kakani, Ramanjulu Sunkar

**Affiliations:** ^1^Department of Biochemistry and Molecular Biology, Oklahoma State University, Stillwater, OK, United States; ^2^Department of Biological Sciences, Faculty of Science, Yarmouk University, Irbid, Jordan; ^3^Cell and Molecular Sciences, The James Hutton Institute, Dundee, United Kingdom; ^4^Informatics and Computational Sciences, The James Hutton Institute, Dundee, United Kingdom; ^5^Department of Plant and Soil Science, Oklahoma State University, Stillwater, OK, United States

**Keywords:** drought tolerance, barley, photosynthesis, proline, RNA-Seq, differential gene expression, alternative splicing

## Abstract

Like many cereal crops, barley is also negatively affected by drought stress. However, due to its simple genome as well as enhanced stress resilient nature compared to rice and wheat, barley has been considered as a model to decipher drought tolerance in cereals. In the present study, transcriptomic and hormonal profiles along with several biochemical features were compared between drought-tolerant (Otis) and drought-sensitive (Baronesse) barley genotypes subjected to drought to identify molecular and biochemical differences between the genotypes. The drought-induced decrease in the leaf relative water content, net photosynthesis, and biomass accumulation was relatively low in Otis compared to Baronesse. The hormonal profiles did not reveal significant differences for majority of the compounds other than the GA20 and the cis-zeatin-o-glucoside (c-ZOG), whose levels were greatly increased in Otis compared to Baronesse under drought. The major differences that emerged from the transcriptome analysis are; (1), the overall number of differentially expressed genes was relatively low in drought-tolerant Otis compared to drought-sensitive Baronesse; (2), a wax biosynthesis gene (CER1), and NAC transcription factors were specifically induced in Otis but not in Baronesse; (3), the degree of upregulation of betaine aldehyde dehydrogenase and a homeobox transcription factor (genes with proven roles in imparting drought tolerance), was greater in Otis compared to Baronesse; (4) the extent of downregulation of gene expression profiles for proteins of the reaction center photosystem II (PSII) (D1 and D2) was low in Otis compared to Baronesse; and, (5), alternative splicing (AS) was also found to differ between the genotypes under drought. Taken together, the overall transcriptional responses were low in drought-tolerant Otis but the genes that could confer drought tolerance were either specifically induced or greatly upregulated in the tolerant genotype and these differences could be important for drought tolerance in barley.

## Introduction

Drought negatively impacts the growth and productivity of many important crops (Bartels and Sunkar, 2005; Kim et al., 2019). Future predictions indicate that drought will worsen, challenging worldwide food security and the needs of an increasing human population (Meza et al., 2020). Indeed, hunger, famine, and malnutrition are expected due to climate change and drought, in addition to other social and political factors (Lobell et al., 2011; Lesk et al., 2016; Cafiero et al., 2018).

Drought is known to disrupt the hormonal balance in plants, which plays an important role in stress tolerance (Peleg and Blumwald, 2011; Bielach et al., 2017; Ullah et al., 2018). The enhanced accumulation of Abscisic acid (ABA) is a hallmark of plant response to drought, which in turn controls the stomatal closure to decrease the transpiration under drought (Munemasa et al., 2015; Ullah et al., 2018). Auxins regulate root growth in response to abiotic stresses including drought (Korver et al., 2018). Similarly, the Gibberellin (GA) mediates many responses to drought. GA concentration is reduced, and the DELLA regulators accumulate, which could be attributed to the retarded growth under drought (Colebrook et al., 2014). Cytokinins (CKs) and their metabolism are important in plants' adaptation to different abiotic stresses including drought (Ha et al., 2012; Pavlu et al., 2018). Both positive and negative effects of CKs on drought tolerance were reported (Zwack and Rashotte, 2015). These observations suggest that the fine-tuned hormonal homeostasis during stress conditions plays an important role in plant's response to abiotic stresses.

Barley (*Hordeum vulgare* L.) is the fourth most important crop plant in terms of production and harvested area (Giraldo et al., 2019). It is a relatively drought resistant crop and is cultivated globally in more than 100 countries (Hiei et al., 2014; Giraldo et al., 2019). Barley is considered as an important model system for dissecting drought tolerance in cereals because of its ability to tolerate drought better than the cereals such as rice and wheat. Moreover, it has a reliable genetic and molecular infrastructure (Dawson et al., 2015).

Barley subjected to drought stress has been investigated previously (Ozturk et al., 2002; Diab et al., 2004; Talamé et al., 2007; Guo et al., 2009; de Mezer et al., 2014; Sallam et al., 2019). Drought significantly reduced the net photosynthetic rate, stomatal conductance, and transpiration in barley (Harb and Samarah, 2015; Mejri et al., 2016; Schmid et al., 2016; Hasanuzzaman et al., 2019). Tibetan barley genotypes subjected to drought revealed the importance of ABA-dependent and ABA-independent signaling pathways during drought, while genes linked to photosynthesis appears to be important during recovery from drought (Zeng et al., 2016). Comparison of barley spikelets' responses in drought-sensitive and drought-tolerant lines revealed a role for a set of more recently evolved genes in the tolerant lines (Hübner et al., 2015). A drought-resistant line exposed to drought stress over 30 days showed acclimation to the stress while the gene expression profiles in this genotype did not differ compared to a drought-sensitive cultivar (Cantalapiedra et al., 2017). Studies also indicated that maintaining a low background expression of drought tolerance related genes under mild stress allows barley to respond more quickly with the onset of the drought stress (Janiak et al., 2019). Furthermore, recent studies underscored the importance of AS in drought-responsive gene expression in barley (Cantalapiedra et al., 2017). By and large, these reports reveal a complex interaction between multiple mechanisms and processes that differ between genotypes/landraces, tissue analyzed and duration of the stress (Hübner et al., 2015; Zeng et al., 2016; Cantalapiedra et al., 2017; Wang et al., 2018). They also highlight the importance of analyzing additional contrasting genotypes to better understand the drought tolerance processes in barley. Deep large-scale transcriptome sequencing allows expression at an individual gene transcript level to be monitored. New quasi-mapping programs (kallisto, salmon) facilitate rapid and highly accurate measurement of transcript level expression but require a comprehensive and accurate reference transcriptome. A first version reference transcript dataset for barley (BaRTv1.0) has recently been established that facilitates measurement of dynamic reprogramming of gene expression in barley and captures post-transcriptional regulation (Rapazote-Flores et al., 2019). In this study, drought-tolerant (Otis) has been compared with drought-sensitive (Baronesse) to identify biochemical and molecular differences associated with differential sensitivities. The drought-responsive RNA-Seq analysis revealed a greater number of differentially expressed genes in Baronesse than in Otis. Interestingly, several genes with proven roles in drought tolerance such as NAC genes, wax biosynthesis gene (CER1), a beta-expansin, and Armadillo (ARM) repeat superfamily were only induced in Otis but not in Baronesse. By contrast, the degree of inhibition of genes associated with PSII (D1 and D2) was much stronger in Baronesse. Furthermore, AS was also found to differ between the genotypes under drought.

## Materials and Methods

### Plant Material

Seeds of Otis and Baronesse genotypes were obtained from Dr. Harold Bockelman, National Small Grains Collection (NSGC), U.S. Department of Agriculture - Agricultural Research Service, Aberdeen, Idaho, USA. Otis is a two-rowed, spring feed barley with high growth and yield in drylands (Mornhinweg et al., 2009). This genotype was developed for growth in dry environments and released by Colorado State University in 1951. Baronesse is a two-rowed, spring, feed barley cultivar that was donated by Peterson Seed Company Incorporation to the NSGC in 1993.

### Growth and Relative Water Content Measurements

Barley seeds were germinated on moist papers and kept in darkness at 24°C. After 3 days, seedlings were transferred to 19 × 13.5 × 17 cm plastic pots filled with BM1 potting medium (peat moss (75–85%), vermiculite, perlite and wetting agent) (Berger, Canada). To ensure both genotypes experienced the same level of drought stress during the treatment, two seedlings of each genotype were transferred to the same pot. The plants were grown in a growth chamber maintained at 25/17°C (day/night temperature), 14/10 h (day/night cycle), 400 μmole m^−1^ s^−1^ light intensity and 50% humidity. Barely plants were fertilized twice (first fertilization was 2 days after seedling transfer to pots, and the second was 10 days after the first fertilization) with Miracle-Gro® Water-Soluble All-Purpose Plant Food (Scotts Miracle Gro, USA). Drought treatment was initiated at the tillering stage Z21 (Zadoks scale) (Zadoks et al., 1974) at which the pots were divided into two groups: the control (well-watered) group and the drought-treated group. For the control group, plants were watered every other day. For imposing drought, watering was withheld, and plants were allowed to experience progressive drought (pDr). For the determination of growth, only main shoot (the shoot that appeared before the tillering stage) was chosen because the differences in the number of tillers among the individual plants of the same genotype varied. After 7 days of pDr, the leaf relative water content (LRWC) was calculated as described (Schonfeld et al., 1988) LRWC% = (Fresh weight-Dry weight)/ (Turgid weight-Dry weight) X 100.

### Gas Exchange Measurements

For assessing photosynthesis-associated parameters, after 5 days of pDr (initial wilting), net photosynthetic rate, stomatal conductance, internal CO_2_ concentration, and transpiration rate of the control and the drought-treated plants were measured on the youngest fully expanded leaf of the control and drought-treated plants (8 plants of each genotype per treatment) using LICOR 6400XT (LI-COR Inc., NE, USA). The following conditions were set for LICOR measurements: flow rate of 300 mmol s^−1^, CO_2_ at 400 mmol, leaf temperature 25°C, and relative humidity of 50%.

### Proline Content

Proline was analyzed according to Carillo and Gibon (2011). Fresh samples from the youngest fully expanded leaf were homogenized using one ml extraction solution (70 ethanol: 30 water). Then, a volume of 100 μl of the extract was added to 200 μl of the reaction mixture (1% (w/v) ninhydrin, 60% (v/v) acetic acid, and 20% (v/v) ethanol). The reaction was kept in a boiling water bath for 20 min, and then kept on ice for stopping the reaction. The absorbance of the reaction mixture was measured at 520 nm.

### Malondialdehyde Accumulation

Oxidative stress was determined by quantification of the malondialdehyde (MDA) levels (Heath and Packer, 1968) with some modifications. Samples of known fresh weight of the youngest fully expanded leaf were collected and snap frozen in liquid nitrogen. The samples were homogenized in 1 ml of 0.1% (w/v) trichloroacetic acid (TCA). Then, they were centrifuged at 4,100 rpm for 10 min. About 100 μl of the supernatant was added to 400 μl of 0.5% (w/v) thiobarbituric acid in 20% (w/v) TCA and the homogenates were boiled at 95°C for 30 min and the reaction was stopped by cooling the tubes on ice. The reaction mixture was centrifuged, and the absorbance was read at 532 nm and 600 nm. After subtracting the non-specific absorbance at 600 nm, the MDA concentration was determined by its extinction coefficient of 155 mM^−1^ cm^−1^.

### Hormonal Profiling

After 5 days of pDr (the initial wilting stage), three biological replicates (ten leaves from 10 different plants were used for each biological replicate) of the youngest fully expanded leaves of the control and the drought-treated plants were collected, snap frozen in liquid nitrogen and kept at −80°C. The frozen samples were lyophilized and used for hormonal analysis. The levels of major hormones and their metabolites were quantified using UPLC ESI-MS/MS by the National Research Council of Canada (Saskatchewan, Canada). The analyzed hormones and metabolites are: cis-abscisic acid (ABA), abscisic acid glucose ester (ABAGE), dihydrophaseic acid (DPA), phaseic acid (PA), 7'-hydroxy-abscisic acid (7'OH-ABA), neo-phaseic acid (neo-PA), trans-abscisic acid (t-ABA), gibberellin 1 (GA1), GA3, GA4, GA7, GA8, GA9, GA19, GA20, GA24, GA29, GA34, GA44, GA51, GA53, indole-3-acetic acid (IAA), N-(indole-3-yl-acetyl)-aspartic acid (IAA-Asp), N-(indole-3-yl-acetyl)-glutamic acid (IAA-Glu), N-(indole-3-yl-acetyl)-alanine (IAA-Ala), N-(indole-3-yl-acetyl)-leucine (IAA-Leu), indole-3-butyric acid (IBA), (trans) zeatin-O-glucoside (t-ZOG), (cis) zeatin-O-glucoside (c-ZOG), (trans) zeatin (t-Z), (cis) zeatin (c-Z), dihydrozeatin (dhZ), (trans) zeatin riboside (t-ZR), (cis) zeatin riboside (c-ZR), dihydrozeatin riboside (dhZR), isopentenyladenine (iP), isopentenyladenosine (iPR), and kinetin (KIN).

### Statistical Analysis

The morphological, physiological, and biochemical data were analyzed using Student's *t*-test, 2-tailed distribution, and type 3 (2-sample unequal variance) (Excel, Microsoft, USA). A difference in means at value < 0.05 was considered significant.

### RNA Sequencing

Three biological samples per genotype per treatment were collected from the youngest fully expanded leaf of 10 plants/sample from the control and the drought treated plants after 5 days of drought (initial wilting stage). The total RNA was extracted following the standard TRIzol method. The RNA integrity was checked with Agilent Technologies 2100 Bioanalyzer (Agilent Technologies, California, USA). Poly(A) tail-containing mRNAs were purified using oligo-(dT) magnetic beads with two rounds of purification. The purified poly(A) RNA was fragmented, and the library was constructed by synthesizing first strand cDNA, followed by second strand cDNA with dUTP, end repair, 3‘ adenylation, adaptor ligation, Uracil-DNA-Glycosylase (UDG) treatment, and PCR. Quality analysis and quantification of the sequencing library were performed using Agilent Technologies 2100 Bioanalyzer High Sensitivity DNA Chip. Paired-ended sequencing was performed on Illumina's NovaSeq 6000 sequencing system (LC Sciences, TX, USA). To remove the reads that contained adaptor contamination, low quality bases and undetermined bases in the sequenced RNA-seq libraries, Cutadapt (Martin, 2011) and perl scripts developed in house were used. Then, sequence quality was verified using FastQC (http://www.bioinformatics.babraham.ac.uk/projects/fastqc/).

### Differential Expression and Differential Alternative Splicing Analyses

The RNA-seq data had 4 treatment groups: Otis, drought treatment (OD) Otis, watered treatment (OW); Baronesse drought treatment (BD) and Baronesse, watered treatment (BW) and each had 3 biological replicates (12 samples in total). Transcript quantifications were generated using Salmon (Patro et al., 2017) and the Barley transcriptome BARTv1.0-QUASI (https://ics.hutton.ac.uk/barleyrtd/index.html) (Rapazote-Flores et al., 2019). The 3D RNA-seq analysis App was used for differential expression (DE) and differential alternative splicing (DAS) analysis (Calixto et al., 2018; Guo et al., 2019). In the pipeline, expressed transcripts were identified when found in ≥ 2 of the 12 samples with count per million reads (CPM) ≥ 1, which provided an optimal mean-variance trend of the read count distribution. The Trimmed Mean of M-values (TMM) method was used to normalize the gene and transcript read counts to *log*_2_-CPM (Bullard et al., 2010). Limma-VoomWeights method was used for DE and DAS (Law et al., 2014; Ritchie et al., 2015). To compare the expression changes between conditions of experimental design, the contrast groups were set as OD-OW, BD-BW, OW-BW, OD-BD. For DE genes, the *log*_2_ fold change (*L*_2_*FC*) of gene abundance were calculated based on contrast groups and *p*-values of multiple testing were adjusted with Benjamini–Hochberg (BH) to correct for false discovery rate (FDR) (Benjamini and Yekutieli, 2001). A gene was significantly DE in a contrast group if it had adjusted *p* < 0.01 and *L*_2_*FC* ≥ 1. For DAS genes, each individual transcript *L*_2_*FC* were compared to gene level *L*_2_*FC*, which was calculated as the weighted average of *L*_2_*FCs* of all transcripts of the gene. Then *p*-values of individual transcript comparison were summarized to a single gene level *p*-value with an *F*-test. A gene was significantly DAS in a contrast group if it had an adjusted *p* < 0.01 and any of its transcripts had a Δ Percent Spliced (ΔPS) ratio ≥ 0.1 (see Supplementary Report).

### Functional Analysis of the DE Genes

The Venn diagram generator of the Bioinformatics and Evolutionary Genomics lab at Ghent University and VIB, Belgium was used to find the unique and the common DE genes in the four contrast groups (http://bioinformatics.psb.ugent.be/webtools/Venn/).

Gene Ontology Tags were applied to the BaRT transcripts using Protein Annotation with Z-score (PANNZER) (Törönen et al., 2018) to produce GO annotations for 25,906 BaRT genes. GO functional enrichment analysis of the DE genes was done using g:profiler (https://biit.cs.ut.ee/gprofiler/gost) (Raudvere et al., 2019) with reference GO annotation dataset file BART_V_1.gmt (https://ics.hutton.ac.uk/barleyrtd/GO_enrichment.html).

The analysis of transcription factors (TFs) and kinases were performed using iTAK online (Zheng et al., 2016). First, the HORV annotation was retrieved for each of DE BaRT genes. Then, BioMart from Ensembl plants was used to get the protein sequence for each gene using Ensembl plants 47 as the database and *Horduem vulgare* genes (IBSC V2) as the dataset (https://plants.ensembl.org/biomart/martview/31c188c3a5aff85045c3cceb489e5597). Protein sequences of the DE genes were the input for the transcription factor and kinase analysis by iTAK online.

### Quantitative Real-Time (qRT) PCR Analysis

Total RNA was used for cDNA conversion and the qRT PCR reactions were performed using the Light Cycler 96 system (Roche). Each PCR reaction was performed on two independent biological samples with two technical replicates. The relative expression levels of the target genes were calculated using the formula 2-ΔΔCt (Livak and Schmittgen, 2001). Fold change was calculated for the drought treated plants relative to the well-watered control, but for cellulose synthase and phenylalanine ammonia lyase the fold change was also calculated for Otis control relative to Baronesse control. Cyclophilin A (BART1_0-p42566) was used as reference gene for data normalization (Burton et al., 2004). Supplementary Table 1 shows the list of primers used.

## Results

### Morphological and Physiological Changes in Barley Genotypes Under Drought

**Figure 1:**
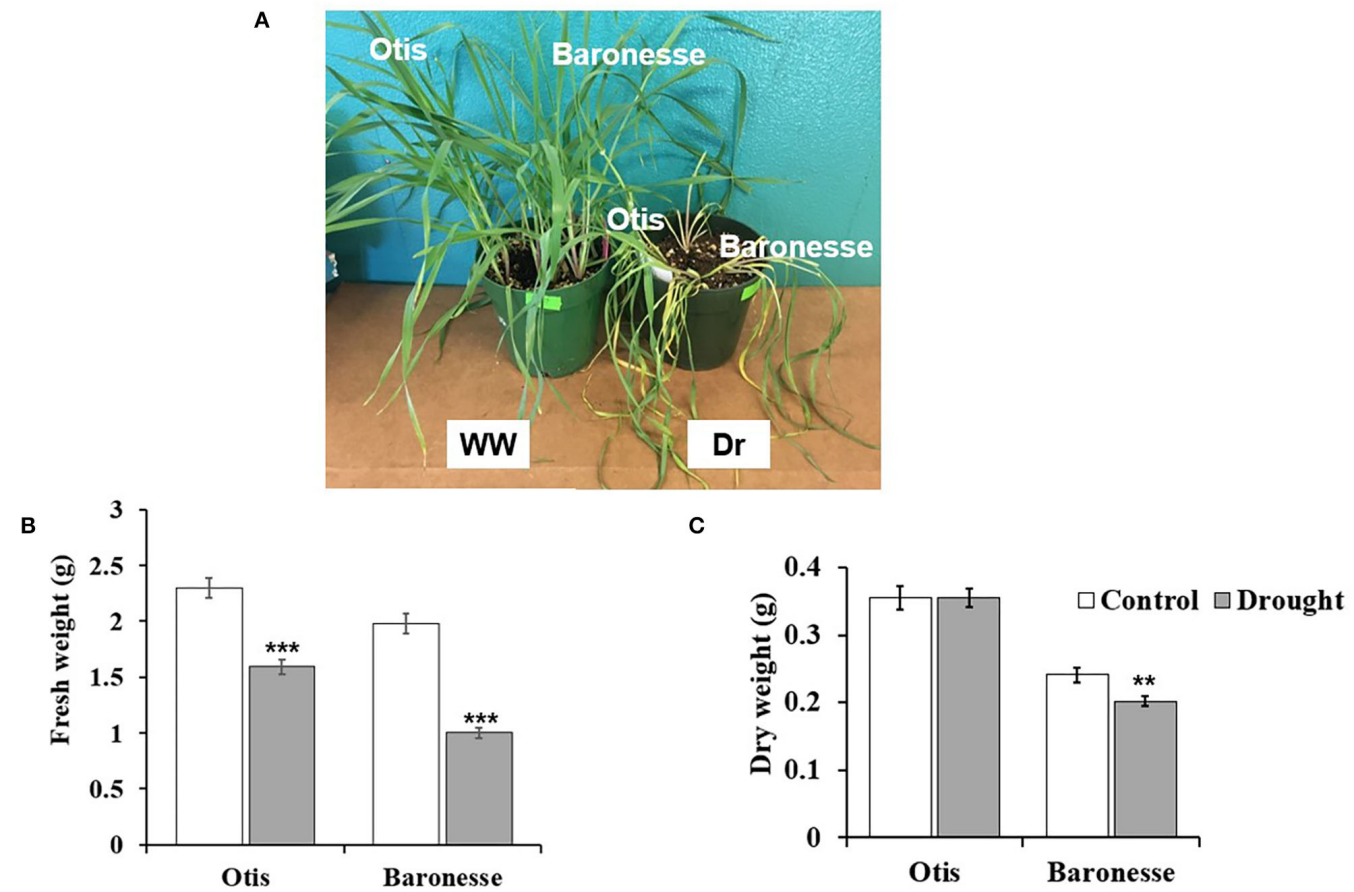
Morphological changes between Otis and Baronesse under drought stress. **(A)** Barley plants of Otis and Baronesse under well-watered (WW) and drought (Dr) conditions. **(B)** Fresh weight (FW) of the main shoot (g). **(C)** Dry weight (DW) of the main shoot (g). Bars represent standard errors of the means. *N* = 10 plants. **P < 0.01 and ***P < 0.001.

Two barley genotypes with expected differences in resistance to drought were deprived of water to examine morphological and physiological differences between the two genotypes. The morphology of Otis and Baronesse after 7 days of drought is shown in Figure 1A. At this stage of drought, the leaves of both genotypes were severe wilting, and yellowing. The fresh weight of the main shoot of Baronesse was reduced by 49.4% of the control compared to 30.6% reduction in Otis (Figure 1B). The dry weight of the main shoot of Baronesse was significantly reduced by 18.7% compared to 0% reduction in Otis (Figure 1C).

**Figure 2:**
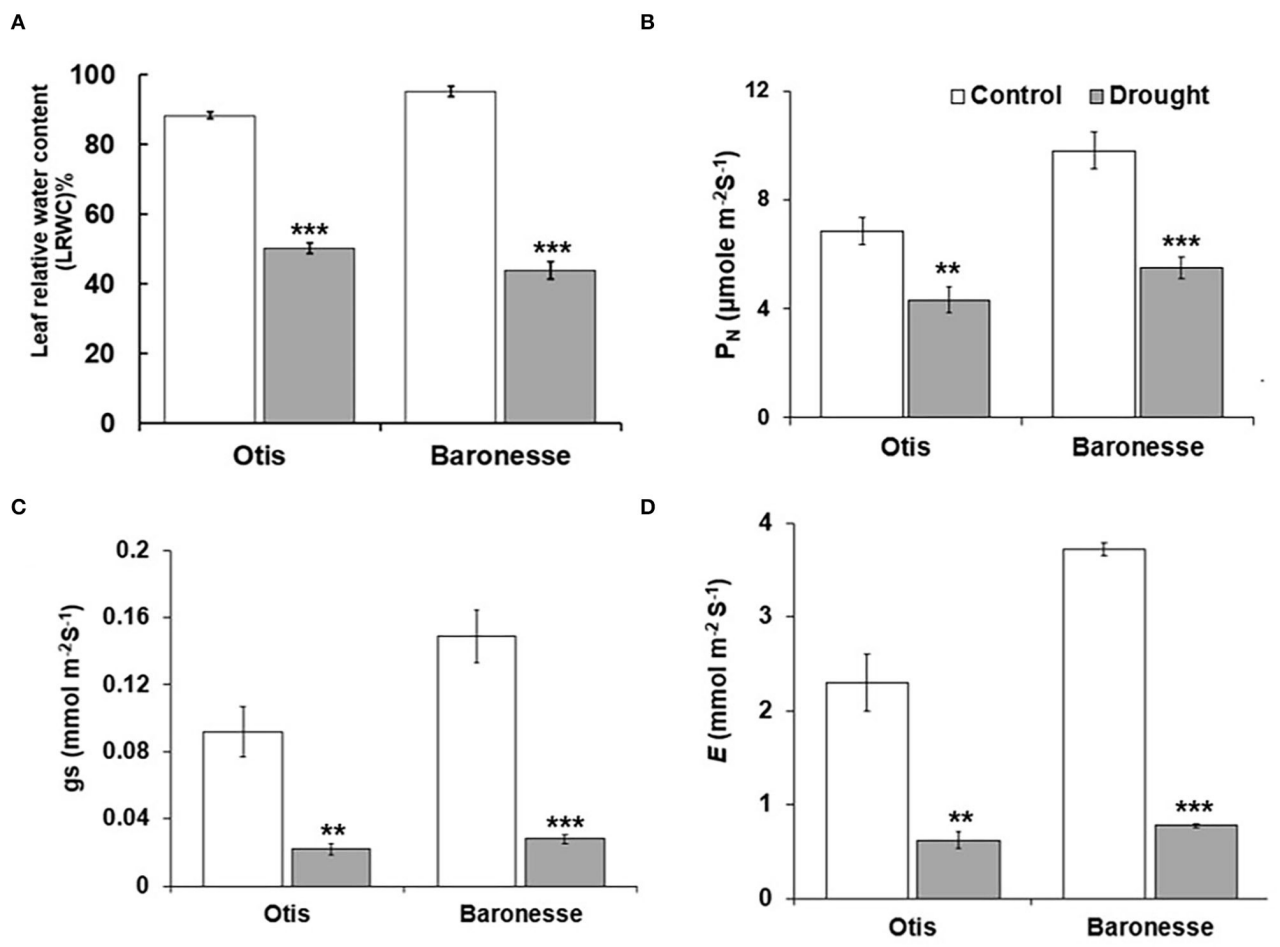
Physiological changes in Otis and Baroness under drought. **(A)** LRWC. **(B)** Net photosynthetic rate (P_N_). **(C)** Stomatal conductance (gs). **(D)** Transpiration rate (*E*). Values are the means of 10 plants for LRWC and 6 plants for photosynthesis measurements. Bars represent standard errors of the means. **P < 0.01 and ***P < 0.001.

In response to drought, the LRWC was significantly reduced in Baronesse compared to Otis (43.9 and 50.2% compared to 95.1 and 88.4% of the control, respectively). At this level of drought, LRWC was 58% of the control in Otis, and 46.2% in Baronesse (Figure 2A). In general, the two genotypes showed a significant decrease in the photosynthetic characteristics [CO_2_ assimilation rate (P_N_), stomatal conductance (gs), and transpiration rate (E)] under drought stress (Figures 2B–D). Under drought, P_N_ was 63% and 56% of the control in Otis and Baronesse, respectively. A similar trend was observed for gs and E (Figures 2C,D). In the drought-treated Otis, the gs and *E* showed 24 and 27% of the control, respectively, whereas these were 19 and 21% of the control, respectively, in Baronesse. Under well-watered conditions, Otis showed significantly less gs and E (0.092 and 2.31 mmol m^−2^ S^−1^) than Baronesse (0.148 and 3.72 mmol m^−2^ S^−1^). Both morphological and physiological tests show that both genotypes respond to the loss of water, but Otis shows greater endurance under these conditions.

### Biochemical and Hormonal Changes in Barley Genotypes Under Drought

**Figure 3:**
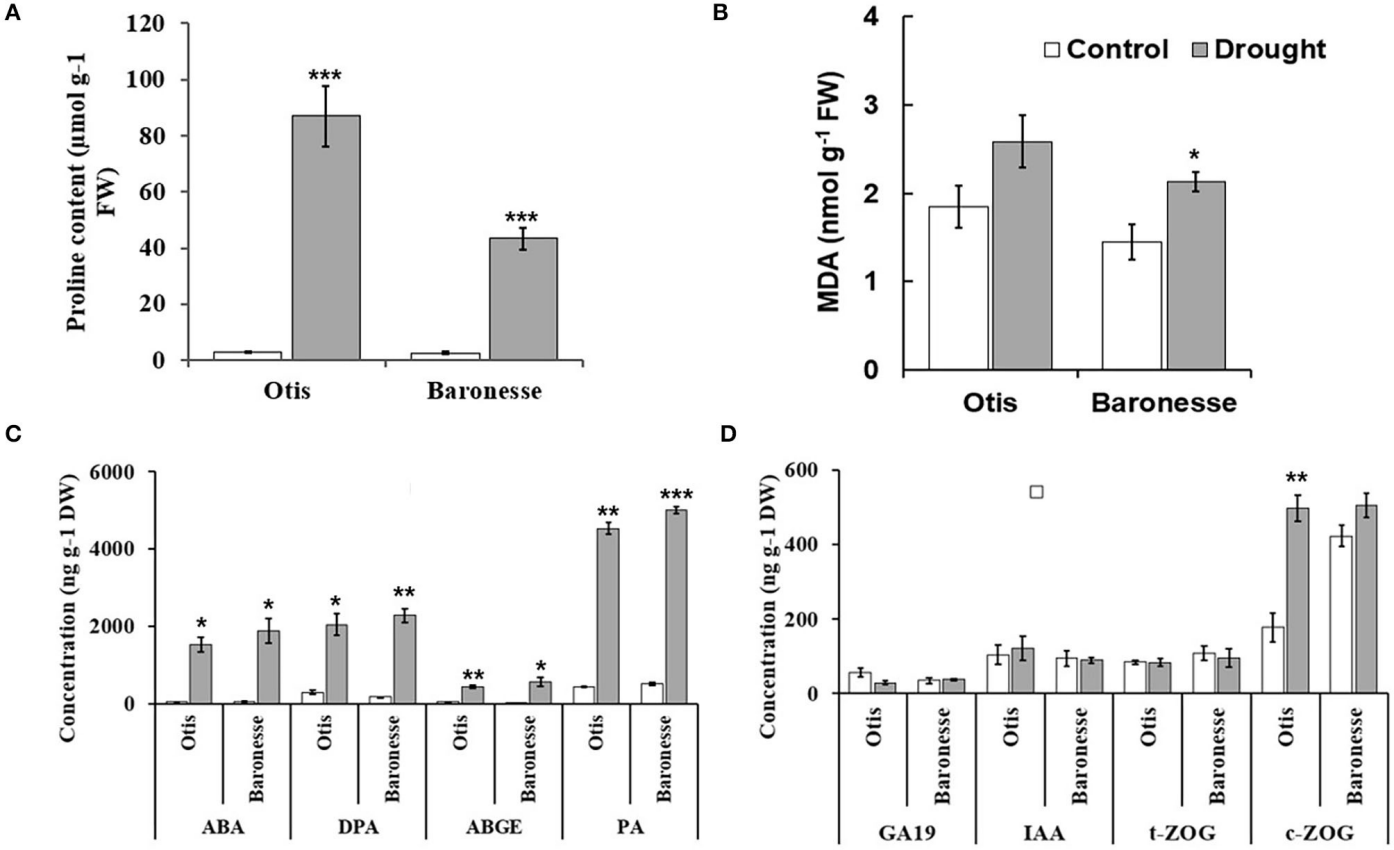
Biochemical and hormonal profiles in Otis and Baronesse under drought stress. **(A)** Proline content. **(B)** Malondialdehyde (MDA) content. **(C)** Concentration of ABA and some of its metabolites (DPA, ABGE, and PA). **(D)** Concentration of Gibberellin 19 (GA19), Indole-3-acetic acid (IAA), trans-zeatin-O- glucoside (t-ZOG), and cis- zeatin-O-glucoside (c-ZOG). Hormones concentrations are the means of 3 biological replicates (each biological replicate is a pool of 10 plants). Bars represent standard errors of the means. *P < 0.05, **P < 0.01, and ***P < 0.001.

The differential responses between the barley genotypes were further assessed using biochemical and hormonal profiles. Proline accumulation was frequently observed in plants subjected to drought. Drought stress significantly increased proline content in the leaves of both genotypes. However, the accumulation found to be higher in Otis (86.94 μmol g-1 FW) compared to Baronesse (43.27 μmol g-1) (Figure 3A). Differential gene expression analysis of proline synthesis and turnover pathway genes further support the accumulation of proline in the leaves under water deficit stress (Supplementary Figure 1). The amount of lipid peroxidation (quantified as MDA) has been often correlated with the degree of stress-induced injury. The concentration of MDA was increased in both the genotypes, although the increase was significant only in the case of Baronesse (Figure 3B).

Changes in the major hormonal groups (ABA, auxins, cytokinins, and GAs) and their metabolites were analyzed in Otis and Baronessse at the initial wilting stage of pDr. The levels of ABA and its metabolites such as DPA, ABGE, and PA were significantly increased in the drought-treated Otis and Baronesse compared to their respective well-watered controls (Figure 3C). The concentration of ABA and its metabolites such as 7‘OH ABA, neo-PA, and t-ABA was not significantly different between the genotypes under both conditions. In the drought-treated Otis, the concentration of ABA, 7‘OH ABA, neo-PA, and t-ABA was 1,528.71, 61.82, 50.69, and 18.22 ng g-1 dry weight (DW), respectively, while their concentration in the drought-treated Baronesse was 1,882.68, 80.96, 60.82, and 22.54 ng g-1 DW, respectively.

The analysis of 14 GAs including GA19 did not reveal significant differences between the genotypes both under well-watered and drought conditions (Figure 3D). However, GA20 was only detected in the drought-treated Otis but not in Baronesse. This observation indicates that the GA20 is specifically induced under drought in drought-tolerant Otis.

Among the auxins, the IAA was detected in both the genotypes under well-watered as well as drought conditions. However, no significant differences were observed between the genotypes under both the conditions (Figure 3D).

The response of cytokinins, specifically t-ZOG accumulation under drought did not reveal significant differences between the genotypes compared to their respective controls (Figure 3D). However, the c-ZOG was significantly increased in the drought-treated Otis but not in Baronesse (Figure 3D). The concentration of c-ZOG was 496.85, and 177.22 ng g-1 DW in the drought-treated and the control plants of Otis genotype, respectively. Similarly, the iPR levels were increased in both the genotypes under drought but the degree of increase was higher in Otis than in Baronesse (3.32 and 2.25 ng g-1 DW in the drought-treated and 1.58 and 1.92 ng g-1 DW in the controls of Otis and Baronesse, respectively).

### Overview of the RNA-Seq Analysis of Drought Response in Two Barley Genotypes

**Figure 4:**
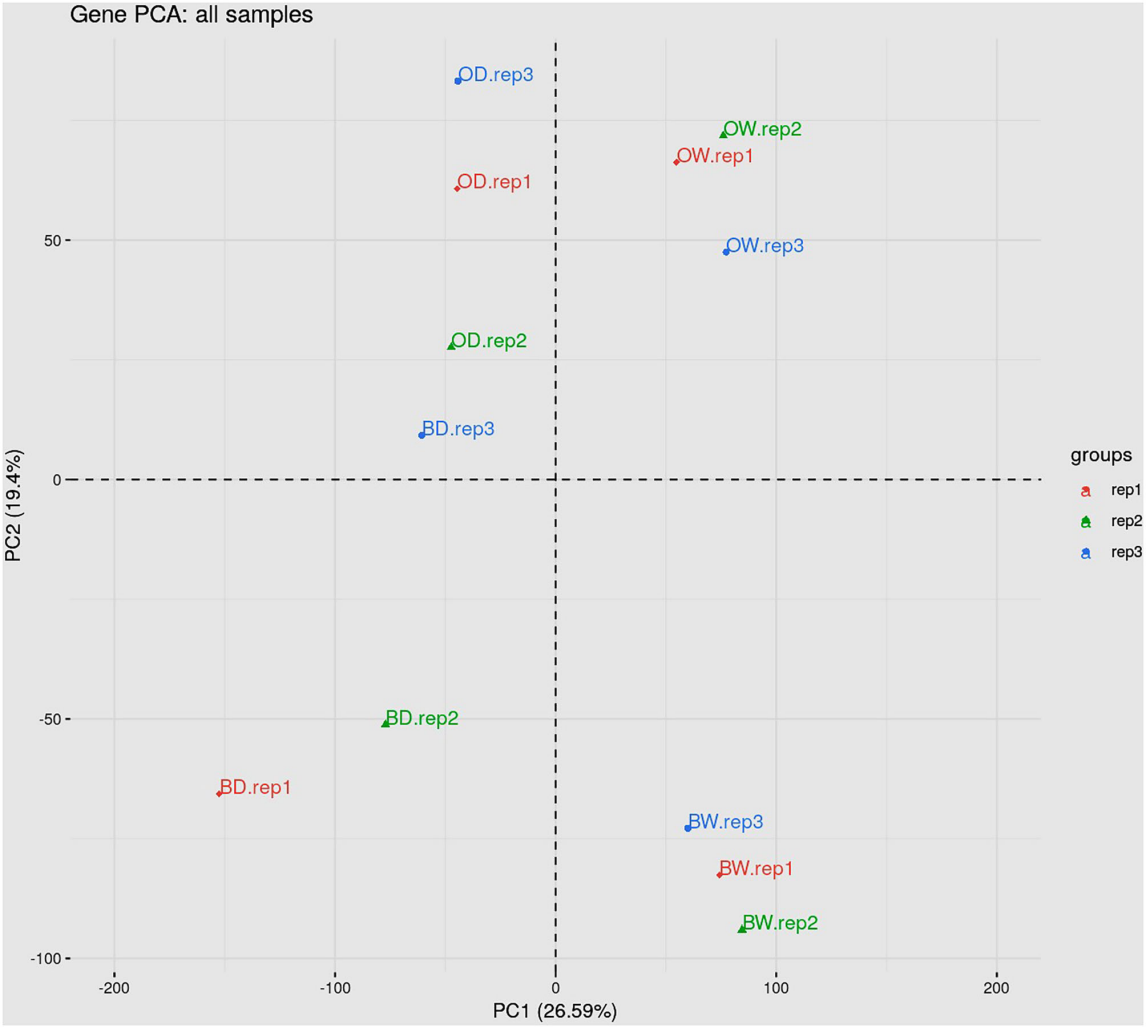
Principal component analysis of the data showing the variation due to genotype and treatment.

**Figure 5:**
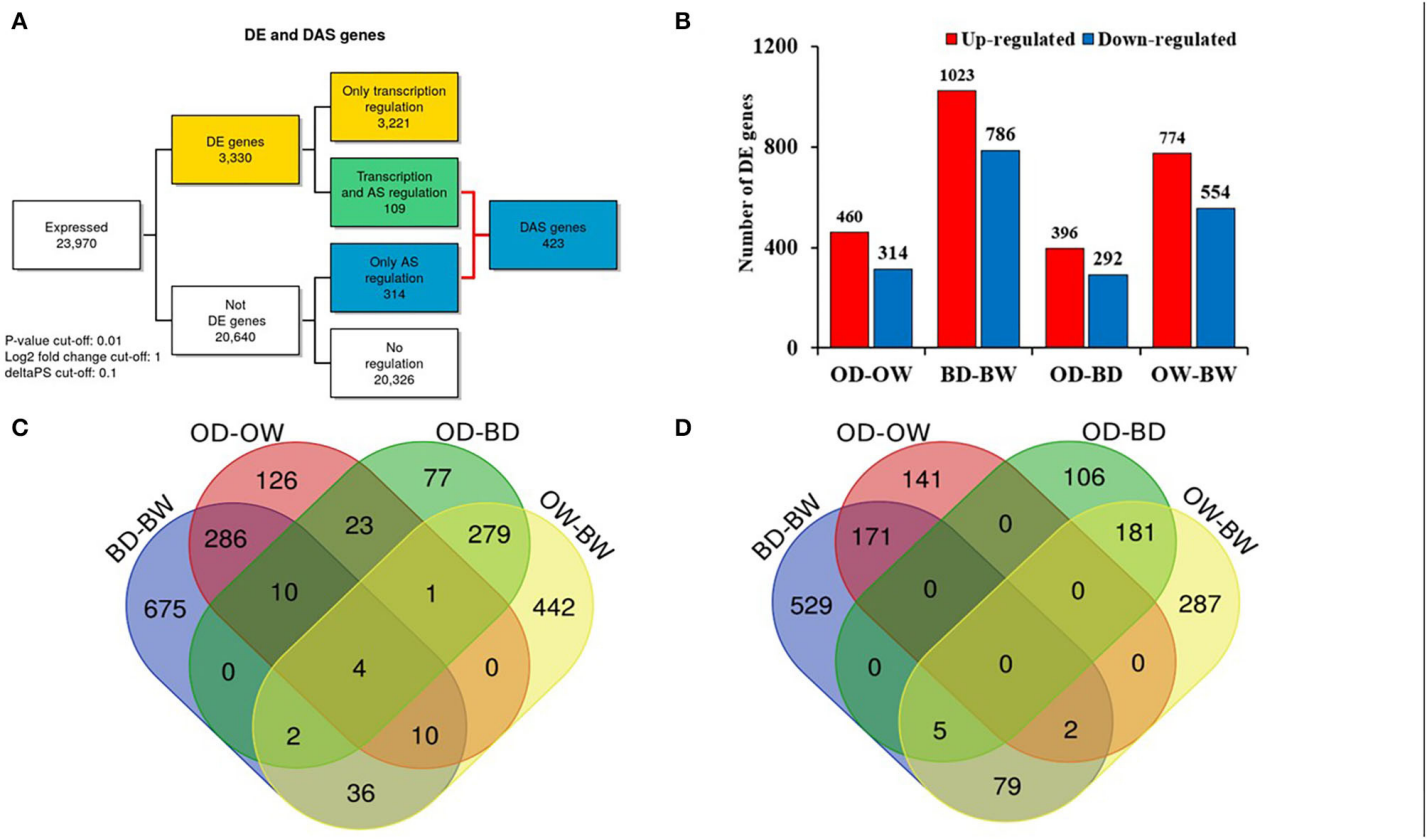
Differential gene and alternative splicing analysis in two barley genotypes under progressive drought. **(A)** Number of genes regulated only by transcription (DE), only by alternative splicing (DAS) and by both transcription and alternative splicing (DE+DAS) across barley contrast groups (OD-OW, BD-BW, OW-BW, and BD-OD). **(B)** Number of up- and down-regulated DE genes in the different contrast groups. **(C)** Venn diagram of the up-regulated genes of the different contrast groups (OD-OW, BD-BW, OD-BD, and OW-BW). **(D)** Venn diagram of the down-regulated genes of the different contrast groups (OD-OW, BD-BW, OD-BD, and OW-BW).

RNA sequencing resulted in at least 40 million paired end reads per sample. The abundance of RNA transcripts in each Otis and Baronesse replicated samples were determined using Salmon and the reference transcript dataset BaRTv1.0. The raw RNA-Seq data has 176,343 transcripts and 59,930 genes After data processing to remove poorly expressed transcripts, there were 57,971 expressed transcripts and 23,970 genes. Principal Component Analysis (PCA) was performed using gene level log_2_ CPM values of the data to visualize RNA-seq data variation between the samples and replicates. The PCA scatter plot shows that replicates of the watered samples of Otis and Baronesse form distinct groups highlighting differences between the two genotypes. The watered samples were also distinct from the water deprived samples which showed less distinct grouping due to some variation between the replicates but both drought treated genotypes remained distinct (Figure 4). Differential gene expression analysis was calculated for four contrast groups (OD-OW, BD-BW, OW-BW, and OD-BD) to compare the differences in gene expression between genotypes Otis and Baronesse (O and B, respectively), and between drought (D) and watered (W) treatments in each of the genotypes. Expression analysis across all the contrast groups revealed a total number of 3,330 significant differentially expressed (DE) genes [adjusted *p* = < 0.01; >2 fold change (log_2_ FC > 1)]; 3,221 genes were regulated at the transcription level (DE), 314 genes were regulated by DAS (adjusted *p* = < 0.01; >10% change in alternative splicing) and 109 genes were regulated by both DE and DAS (Figure 5A). The BD-BW contrast group showed the highest total of DE genes (1,203 up-regulated and 786 down-regulated), and OD-BD showed the lowest number of DE genes (396 up-regulated and 292 down-regulated), indicating that Baronesse showed the greatest transcriptional response to 5 days of drought. Some genes showed a similar up (286 genes) and downregulated (171 genes) expression response in both genotypes to the water deprived conditions (Table 1; Figure 5B). However, both genotypes responded differently to the drought conditions and there were 675 Baronesse genes and 126 Otis genes that were uniquely regulated in each genotype contributing to a different response by these two genotypes to the condition (Figures 5C,D). In addition, each genotype showed differentially expressed genes despite application of the condition (contrast groups OW-BW and OD-BD). These gene expression differences highlight common responses to the deprived water condition but also show that these genotypes differ substantially from each other, which supports the morphological and physiological variation found. Supplementary Tables 2–7 show the detailed results of RNA Seq and gene expression.

**Table 1:** Number of genes that are regulated by differential expression and/or by differential alternative splicing (DAS) in the different contrast groups.

**Contrast**	**DE genes**	**DE only**	**DE and DAS**	**DAS only**
OD-OW	774	773	1	36
BD-BW	1,809	1,806	3	61
OW-BW	1,328	1,280	48	260
OD-BD	688	669	19	131

### Differentially Expressed Genes in Otis and Baronesse Under Drought

The categories of genes enriched in response to water deprivation were determined by performing a GO-enrichment analysis. The top three functional groups that were enriched in the common up-regulated genes between Otis and Baronesse were: response to water deprivation (GO:0009414), cation binding (GO:0043169), and raffinose alpha-galactosidase activity (GO:0052692) (Table 2). The unique up-regulated genes in Otis showed no significantly enriched processes. Whereas, the unique up-regulated genes of Baronesse were significantly enriched for cytosolic part (GO:0044445) and structural constituent of ribosome (GO:0003735) (Table 2).

**Table 2:** GO enrichment of the differentially expressed genes in Otis and Baronesse under drought compared to well-watered control (OD-OW and BD-BW).

**Commonly up-regulated genes between Otis and Baronesse under drought (OD-OW and BD-BW)**					
**Term name**	**Term ID**	**Adjusted** ***P*****-value**	**Term size**	**Query size**	**Intersection size**
Response to water deprivation	GO:0009414	1.42E-07	399	240	21
Cation binding	GO:0043169	0.000373003	75	240	8
Raffinose alpha-galactosidase activity	GO:0052692	0.000416886	33	240	6
Metabolic process	GO:0008152	0.01750248	37	240	5
**Commonly down-regulated genes between Otis and Baronesse under drought (OD-OW and BD-BW)**					
Peptidyl-tyrosine modification	GO:0018212	0.007915027	507	145	13
**Uniquely up-regulated genes BD-BW**					
Cytosolic part	GO:0044445	0.00131172	146	507	14
Structural constituent of ribosome	GO:0003735	0.006559518	1,094	507	44
**Uniquely down-regulated genes BD-BW**					
Protein phosphorylation	GO:0006468	9.99E-12	3,331	422	113
ATP binding	GO:0005524	1.30E-07	6,392	422	163
Plasma membrane	GO:0005886	3.88E-07	3,249	422	99
Protein kinase activity	GO:0004672	1.35272E-05	2,578	422	80

The common down-regulated genes between the genotypes under water deprivation were found to be enriched for peptidyl-tyrosine modification (GO:0018212) (Table 2). The uniquely down-regulated genes of Otis (OD-OW) showed no significantly enriched processes. The top four processes that were enriched in the unique down-regulated genes of Baronesse (BD-BW) are: protein phosphorylation (GO:0006468), ATP binding (GO:0005524), plasma membrane (GO:0005886), and protein kinase activity (GO:0004672).

The BARTV1.0 and HORVU annotations of the 21 genes under the GO term response to water deprivation (GO:0009414) are shown in Table 3. The common up-regulated genes showed known stress responsive genes such as chaperones, annexin, signaling genes (kinases and phosphatases), aquaporin, and transcription factors. The expression level of most of these genes in the drought treated plants of the two genotypes is almost the same, except for a few genes. For example, the log_2_ FC of homeobox-leucine zipper protein ATHB-6 is 3.83 and 4.89 in the drought treated Otis and Baronesse plants, respectively. And the log_2_ FC of betaine aldehyde dehydrogenase was 2.43 and 1.52 in the drought treated Otis and Baronesse, respectively.

**Table 3:** The BART and HORVU annotations of the commonly up-regulated genes under drought.

**BART gene ID**	**BART annotation**	**HORVU gene ID**	**HORVU annotation**	**Log_**2**_ FC OD-OW**	**Log_**2**_ FC BD-BW**
BART1_0-P29927	2C-type protein phosphatase protein	HORVU4Hr1G060370	Protein phosphatase 2C family protein	3.41	3.33
BART1_0-P34164	Molecular chaperone HtpG	HORVU5Hr1G027910	Chaperone protein htpG family protein	1.55	1.44
BART1_0-p07678	Aquaporin protein	HORVU2Hr1G010990	Aquaporin-like superfamily protein	3.11	2.58
BART1_0-P51213	Low quality protein: annexin D2	HORVU7Hr1G037080	Annexin 1	3.08	3.57
BART1_0-P39278	Cold-regulated plasma membrane protein 2	HORVU5Hr1G098190	Cold acclimation protein WCOR413 family	1.69	2.59
BART1_0-P07561	Putative ATP-binding cassette subfamily C member 8	HORVU2Hr1G009580	ABC transporter C family member 14	2.58	2.08
BART1_0-P50224	Leucine-rich repeat, cysteine-containing subtype	HORVU7Hr1G023610	F-box protein MAX2	2.15	1.44
BART1_0-P45680	Homeobox-leucine zipper protein ATHB-6	HORVU6Hr1G061390	Homeobox-leucine zipper protein family	3.83	4.89
BART1_0-P36679	Non-specific serine/threonine kinase protein kinase	HORVU5Hr1G065350	Serine/threonine protein kinase 1	1.78	1.32
BART1_0-P35808	RNA recognition motif domain	HORVU5Hr1G053230	RNA-binding protein 1	1.32	1.17
BART1_0-P47576	Betaine aldehyde dehydrogenase	HORVU2Hr1G070680	Betaine aldehyde dehydrogenase 2	2.43	1.52
BART1_0-P13794	Abscisic stress-ripening protein 2	HORVU2Hr1G092710	Homeobox-leucine zipper protein family	3.17	1.75
BART1_0-P47022	Class I homeodomain-leucine zipper protein 22	HORVU6Hr1G080670	bZIP transcription factor 27	1.49	1.31
BART1_0-P12382	G-box-binding factor 4	HORVU2Hr1G074770	Abscisic stress-ripening protein 3	3.49	3.60
BART1_0-P46765	Signal transduction response regulator	HORVU6Hr1G077070	Histidine kinase 3	1.28	1.15
BART1_0-P37103	Molecular chaperone HtpG	HORVU5Hr1G070720	Chaperone protein htpG family protein	1.65	1.79
BART1_0-P29183	Sucrose synthase	HORVU4Hr1G049500	Sucrose synthase 3	1.28	1.15
BART1_0-P29382	Hexosyltransferase	HORVU4Hr1G052450	Hexosyltransferase	2.79	2.17
BART1_0-P21831	2C-type protein phosphatase protein	HORVU3Hr1G067380	Protein phosphatase 2C family protein	2.98	3.27
BART1_0-P15058	Putative zeaxanthin epoxidase	HORVU2Hr1G106880	Chloroplastic lipocalin	1.18	1.21
BART1_0-P29181	Sucrose synthase	HORVU4Hr1G049500	Sucrose synthase 3	2.03	2.02

The common down regulated genes between Otis and Baronesse were enriched for peptidyl-tyrosine modification (GO:0018212). Under this process 13 genes were down regulated under drought compared to the control. The BART and HORVU annotations of these genes are shown in Table 4. In general, the common-down regulated genes are groups of kinases such as cysteine-rich receptor like protein kinase 5, leucine-rich receptor-like protein kinase family protein isoform 2, and serine/threonine protein kinase. A number of these kinase genes show a much greater down-regulation in expression in Baronesse compared to Otis (Table 4). For example, the Log_2_ FC of LRR receptor-like serine/threonine-protein kinase EFR (BART1_0-p15086) is −1.94 in Otis and −3.18 in Baronesse.

**Table 4:** The BART and HORVU annotations of the common down-regulated genes in barley plants under drought stress.

**BART gene ID**	**BART annotation**	**HORVU gene ID**	**HORVU annotation**	**Log_**2**_FC OD-OW**	**Log_**2**_FC BD-BW**
BART1_0-p16694	Cysteine-rich receptor-like protein kinase 5 (Fragment)	HORVU2Hr1G041380	receptor kinase 1	−2.77	−3.62
BART1_0-p22957	Leucine-rich receptor-like protein kinase family protein isoform 2	HORVU3Hr1G081600	Leucine-rich repeat receptor-like protein kinase family protein	−2.66	−3.33
BART1_0-p16935	Serine-threonine/tyrosine-protein kinase catalytic domain-containing protein	HORVU3Hr1G000350	Protein kinase superfamily protein	−2.14	−3.32
BART1_0-p41113	Serine/threonine-protein kinase	HORVU5Hr1G120420	Receptor serine/threonine kinase, putative	−1.98	−3.25
BART1_0-p15086	LRR receptor-like serine/threonine-protein kinase EFR	HORVU2Hr1G107180	Leucine-rich receptor-like protein kinase family protein	−1.94	−3.18
BART1_0-p10197	Protein serine/threonine kinase	HORVU2Hr1G042210	Serine/threonine-protein kinase	−1.68	−2.24
BART1_0-p16723	ATP binding protein	HORVU2Hr1G124530	Protein kinase superfamily protein	−1.66	−2.12
BART1_0-p38965	Serine/threonine-protein kinase HT1	HORVU5Hr1G094510	Protein kinase superfamily protein	−1.60	−1.49
BART1_0-p10214	Protein serine/threonine kinase	HORVU2Hr1G042220	Serine/threonine-protein kinase	−1.58	−1.47
BART1_0-p45360	LRR receptor-like serine/threonine-protein kinase EFRprotein	HORVU6Hr1G057240	Leucine-rich repeat receptor-like protein kinase family	−1.37	−1.22
BART1_0-p16670	Protein kinase	HORVU2Hr1G125210	Receptor kinase 1	−0.133	−1.21
BART1_0-p13090	Inactive LRR receptor-like serine/threonine-protein kinase BIR2	HORVU2Hr1G084260	Receptor-like protein kinase 4	−1.23	−1.07
BART1_0-p51631	Transmembrane receptor protein serine/threonine kinase	HORVU7Hr1G043150	Protein kinase superfamily protein	−1.14	−1.04

### The Most Highly Regulated Genes Under Drought in Genotype-Dependent Manner

Differential gene expression analysis of the RNA-seq data identified highly induced or reduced genes as a response to drought. Both genotypes showed a different gene responding highly to the condition. In Otis, CER1 protein (BART1_0-p02677), Triticum beta-expansin (BART1_0-p22302), multidrug/pheromone exporter, ABC superfamily (BART1_0-p46064), Armadillo (ARM) repeat superfamily protein (BART1_0-p34106), STAM-binding protein (BART1_0-p13576), jasmonate induced protein (BART1_0-p25925), and NAC-type transcription factor (BART1_0-p58823) showed an expression level of > 5 Log_2_ FC. In Baronesse, the top up-regulated genes with Log_2_ FC > 7.5 were: peptidyl-prolyl cis-trans isomerase (BART1_0-p44951), monooxygenase (BART1_0-p00176), late embryogenesis abundant protein-like (BART1_0-p38756), dehydrin (BART1_0-p23589), late embryogenesis abundant (BART1_0-p47280), rRNA N-glycosylase (BART1_0-p31866), late embryogenesis abundant protein (BART1_0-p48484), and asparagine synthetase [glutamine-hydrolyzing] (BART1_0-p35535) (Supplementary Table 8).

The most significant down-regulated genes in Otis were catalytic genes such as NADPH-hemoprotein reductase (BART1_0-p22029), myrcene synthase, chloroplastic (BART1_0-p56454), glucan endo-1,3-beta-glucosidase 13 (BART1_0-p06463), peroxidase (BART1_0-p08311), and O-acyltransferase WSD1 (BART1_0-p05934). In Baronesse, 6 of the most significant down-regulated genes were kinases such as L-type lectin-domain containing receptor kinase IX.1 (BART1_0-p48873), putative receptor protein kinase ZmPK1 (BART1_0-p21390), and Cysteine-rich receptor-like protein kinase 25 (BART1_0-p06472). Interestingly, aquaporin (BART1_0-p57239), transcription factor MYB4 (BART1_0-p45446), and nicotianamine synthase (BART1_0-p47748) are among the most highly down-regulated genes in Baronesse (see Supplementary Table 9).

### Photosynthesis Genes Were Repressed Under Drought

The expression levels of several photosynthesis related genes were significantly down regulated in both the genotypes, indicating a general suppression of photosynthesis under drought. In drought-treated Otis, the expression level of photosystem II protein D1 (BART1_0-p16339), NAD(P)H-quinone oxidoreductase subunit 2, chloroplastic (BART1_0-p59777), ferredoxin-dependent glutamate synthase, chloroplastic-like (BART1_0-p10355), ATPase subunit IV (BART1_0-p59370), and proton-transporting ATP synthase activity (BART1_0-p26862) were significantly downregulated. Likewise in Baronesse, the abundances of PSII protein D1 (Fragment) (BART1_0-p60027), PSII D2 protein (Fragment) (BART1_0-p16337), NAD(P)H-quinone oxidoreductase subunit 2, chloroplastic (BART1_0-p44871), photosynthetic NDH subunit of subcomplex B (BART1_0-p46676), NADH-plastoquinone oxidoreductase subunit 5 (BART1_0-p03209), and ATP synthase CF1 alpha subunit, chloroplastic (BART1_0-p60215) were significantly down regulated under drought.

### The Differentially Expressed Kinases in the Two Genotypes Under Drought

**Figure 6:**
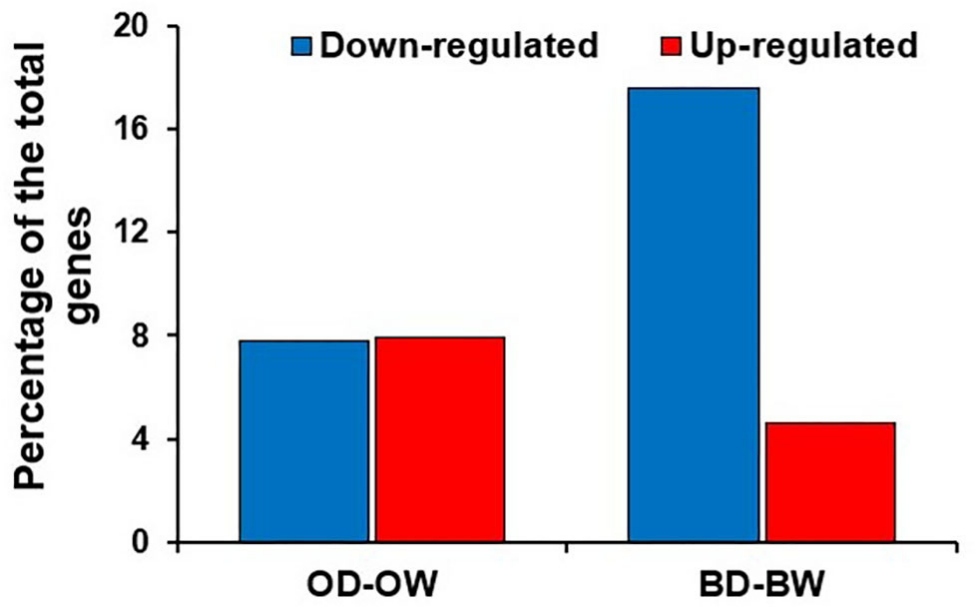
Percentage of DEGs encoding kinases in Otis and Baronesse under drought.

In plants, the kinases represent one of the largest category of genes which play significant roles in response to stress conditions. Differential gene expression analysis revealed that the kinases are the highest number of down-regulated genes in Baronesse under drought (Figure 6). Kinases account for 17.6 and 4.6% of down- and up-regulated genes in Baronesse, respectively, while the differentially regulated kinases represent 8% each for the upregulated and downregulated categories in Otis.

The DE kinases in the two genotypes are shown in Table 5; Supplementary Tables 10–13. In the down-regulated group of genes from Baronesse, the dominant family-subfamily was receptor like kinase-Pelle (RLK-Pelle). In this family/subfamily, 14 types of kinases were significantly down regulated more than 2-fold, including 10 RLK-Pelle-DLSV, 8 RLK-Pelle-WAK, 8 RLK-Pelle-L-LEC, and 7 RLK-Pelle-SD-2b. Baronesse also showed 7 RLK-Pelle genes up-regulated more than 2-fold. Otis showed 12 categories of kinases regulated under reduced water. For example, two Ca2+/calmodulin-dependent protein kinases (CAMK) (OST1L and CAMKL-CHK1) were significantly down-regulated while CAMK-CAMKL-CHK1 was uniquely up-regulated in Otis. In addition, two different categories of plant specific tyrosine kinase like (TLK) genes were significantly up regulated in the two genotypes under drought conditions.

**Table 5:** The differentially expressed kinases in Otis and Baronesse under drought stress.

**OD-OW Down**	**OD-OW Up**	**BD-BW Down**	**BD-BW Up**
CAMK (Ca2+/calmodulin-dependent protein kinase) _OST1L (Open stomata-like kinase)	CAMK_CDPK (calcium-dependent protein kinases)	CAMK_CAMKL-CHK1	CAMK_CDPK
CAMK_CAMKL-CHK1 (CAMK-Like, Checkpoint Kinase 1)	CAMK_CAMKL-CHK1	WNK_NRBP [With No Lysine (K)' kinases and nuclear receptor binding protein (NRBP)]	RLK-Pelle_RLCK-VIIa-1
RLK-Pelle_RLCK-VIIa-2 (Receptor Like Cytoplasmic Kinase-VIIa-2)	CMGC_MAPK	PEK_GCN2 (Pancreatic eukaryotic initiation factor-2alpha kinase, general control non-derepressible)	RLK-Pelle_DLSV
RLK-Pelle_WAK (Wall Associated Kinase)	RLK-Pelle_DLSV (DUF26, SD-1, LRR-VIII and VWA, a moss-specific new RLK subfamily)	ULK_ULK4 (Unc-51 Like Kinase 4)	RLK-Pelle_CrRLK1L-1 (Catharanthus roseus RLK1-like)
RLK-Pelle_LRR-XI-2 (Leucine-rich repeat-XI-2)	RLK-Pelle_WAK	NEK [Mitotic Kinase family, also known as NRK (NimA-Related Kinase, based on Aspergillus NimA)]	RLK-Pelle_RLCK-Os
	RLK-Pelle_LRR-V	RLK-Pelle_WAK	RLK-Pelle_RLCK-V
	TKL-Pl-4 (Tyrosine kinase like Plant-specific 4)	RLK-Pelle_SD-2b (S Domain 2b)	RLK-Pelle_L-LEC
		RLK-Pelle_LRR-Xa	RLK-Pelle_PERK-1 (Plant External Response Like Kinase 1)
		RLK-Pelle_LRR-VIII-1	TKL_Gdt (growth-differentiation transition)
		RLK-Pelle_L-LEC (L-type lectin)	
		RLK-Pelle_LRR-XII-1 (Leucine-rich repeat-XII-1)	
		RLK-Pelle_RLCK-Os (Receptor Like Cytoplasmic Kinase-Os)	
		RLK-Pelle_LRR-Xb-1	
		RLK-Pelle_LRK10L-2 (LRK10-like kinase type 2)	
		RLK-Pelle_DLSV	

### Differentially Expressed Transcription Factors in the Two Genotypes Under Drought

Transcription factors (TFs) are key regulatory genes that coordinate regulation of plant development and conditional responses to a variety of stresses including drought. The number of differentially expressed TFs was higher in Baronesse (52 genes) compared to Otis (8 genes) (Supplementary Tables 14–17). In Otis, two bZIP and one NAC TFs were significantly down-regulated while mainly NAC TFs were up-regulated. In Baronesse plants under drought stress, 4 out of 21 down-regulated TFs were WRKY, 3 MYB and 3 bZIP domain TFs were down-regulated too. The up-regulated TFs in drought-stressed Baronesse were 3 GATA, 2 NAC domain, 2 bZIP, 2 MYB, nuclear factor Y subunit B, PLATZ, trihelix, and ethylene-responsive transcription factor 5 TFs.

### Altered Expression of Chromatin Remodeling and Epigenetics-Associated Genes Under Drought

Epigenetic mechanisms are involved in the plant's transcriptional response to environmental stresses such as drought. Baronesse showed the greatest transcriptional response to water depravation and showed regulation of chromatin remodeling genes. One histone methyl transferase (SET) (BART1_0-p53128) and 2 Snf2-family ATPases (SNF2 chromatin remodeler) (BART1_0-p18056, BART1_0-p51557) were significantly down regulated while two SET (BART1_0-p38488, and BART1_0-p46523) and 1 GCN5-related N-terminal acetyltransferase (GNAT) (BART1_0-p31567) genes were up-regulated.

### Differential Alternative Splicing Under Drought Stress

**Figure 7:**
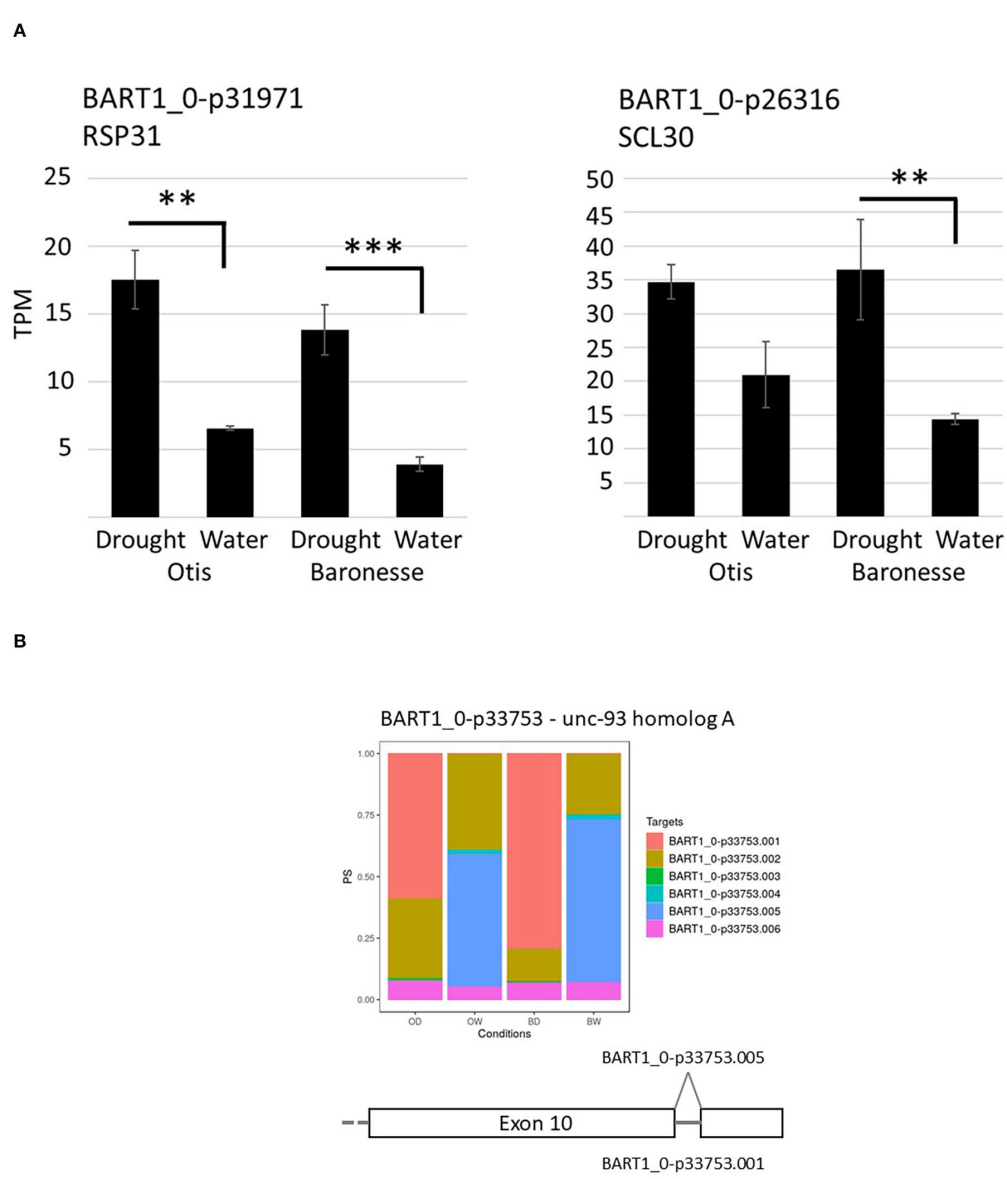
Expression analysis of AS associated genes. **(A)** Mean differential gene expression of barley orthologs of SR splicing factors, RS31 and SCL30. Histograms show expression levels in transcripts per million (TPM) for watered and drought-treated Otis and Baronesse. Standard errors are the result of three biological repeats. Adjusted *p* values on log_2_ FC: ** < 0.005; *** < 0.001. **(B)** Expression analysis of UNC93-A AS transcripts. Each gene transcript is represented by a different color. Each histogram bar represents an individual genotype under a specific condition. From left to right is Otis drought (OD), Otis watered (OW), Baronesse drought (BD) and Baronesse watered (BW).

Serine and arginine-rich (SR) proteins are a group of highly conserved alternative splicing factors that have a role in regulating AS, changing the proportions of gene transcript isoforms under different plant stresses (Duque, 2011). Differential gene expression analysis identified barley orthologs of splicing factor RS31 (BART1_0-p31971; HORVU4Hr1G088790) and SC35-like splicing factor SCL30 (BART1_0-p26316; HORVU1Hr1G043200) genes that were up-regulated in response to water deprivation. RS31 showed a 2.7-fold increase in Otis and 3.6-fold in Baroness, while SCL30 showed a 1.7-fold increase in Otis and 2.5-fold increase in Baronesse in response to the drought stress (Figure 7A). Gene expression analysis using the barley reference transcript dataset allowed quantification of individual transcript isoforms and to determine significant DAS events using the 3D RNA-seq App (Rapazote-Flores et al., 2019). To identify significant DAS genes, expression changes of a log2 fold change between gene transcripts were determined along with an adjusted *p* < 0.01 and a minimum 0.1 (10%) change in the proportion of spliced transcripts (Δ Percent Spliced – ΔPS). Across the two genotypes under watered and drought conditions 423 genes were detected that showed significant changes in transcript isoforms across the different genotypes and conditions and 109 of these genes were regulated by both transcription and AS such that 314 genes were uniquely regulated by AS, with no overall significant change in gene expression (Figure 5A). Pair-wise comparisons of Otis and Baronesse's response to drought stress showed only 37 and 61 significant DAS genes, respectively, and only 6 genes were common between the two genotypes (Table 1; Supplementary Figure 2; Supplementary Tables 18–22). Of the 6 genes showing significant changes in AS in both genotypes, BART1_0-u33753 (HORVU5Hr1G021770) has similarity to unc-93 homolog A, a positive regulator of abiotic stress tolerance in Arabidopsis (Xiang et al., 2018). This showed a complete reversal of the most abundant transcript BART1_0-u33753.005 in the watered samples of both genotypes to the BART1_0-u33753.001 transcript which was most abundant in drought samples (Figure 7B). This complete switch in transcript processing does not affect the protein coding sequence but results in the retention of an intron in the 3'UTR. GO enrichment analysis did not find any enrichment of GO terms, due to the broad range of different types of genes affected by AS genes and low number of AS genes found. These studies suggest that alternative splicing is less frequently affected under drought compared to other abiotic stresses. The results here also show genotype-specific differences in DAS responses under drought.

### Validation of the RNA-Seq Profiles Using RT-qPCR

**Figure 8:**
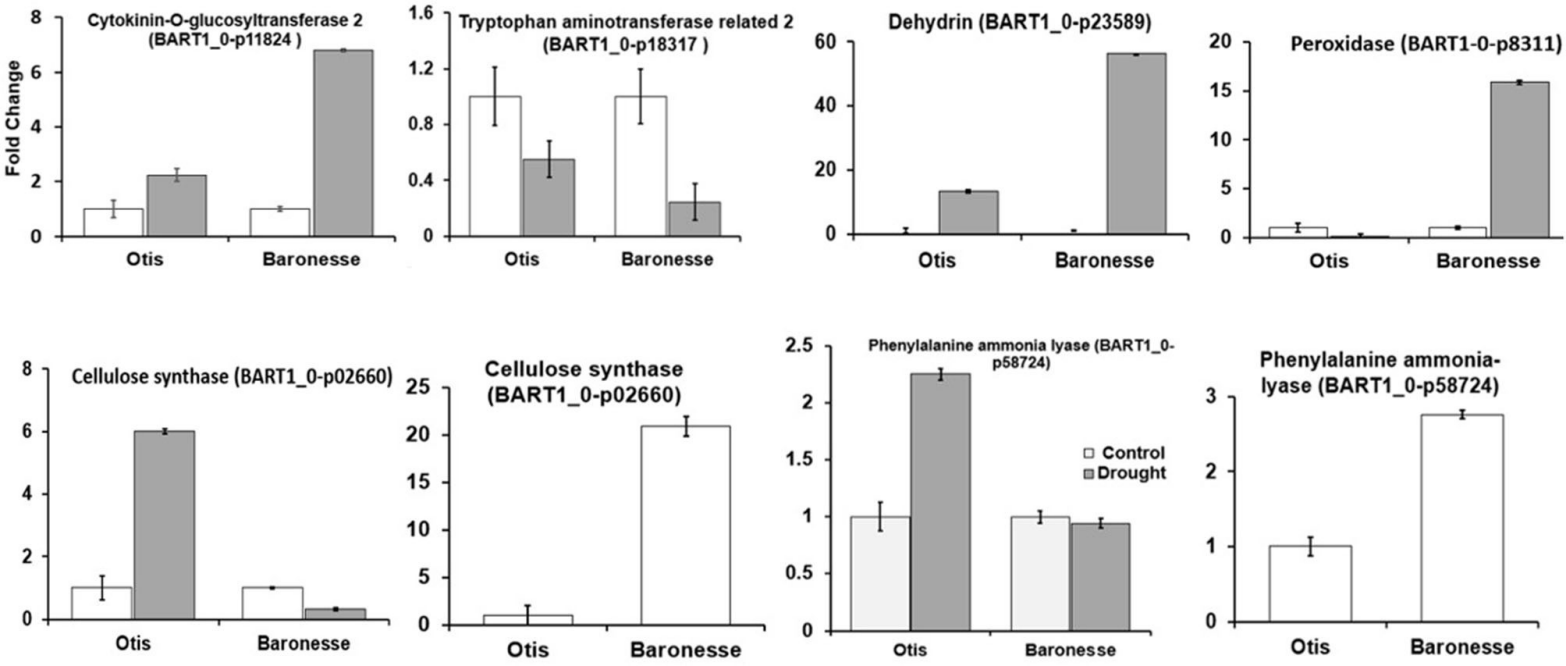
Quantitative real-time PCR validation of RNA-seq data for selected genes.

We used qRT-PCR and validated the gene expression profiles of several genes (Figure 8). For example, the cytokinin-o-glucosyltransferase 2 (BART_0-p11824) was up-regulated under drought stress in both the genotypes (OD-OW and BD-BW showed 1.69- and 1.30-fold change (RNA Seq) and 2.23 and 6.81 (qPCR), respectively). Tryptophan aminotransferase related 2 (BART1_0-p18317) was down-regulated in Baronesse under drought (-2.67 (RNA Seq) and 0.24-fold change (qPCR). Cellulose synthase was downregulated in Otis plants under control conditions compared to Baronesse plants [-2.99 (RNA Seq) and−19.87 (qPCR]. But it was up regulated in Otis under drought stress compared to Baronesse. Phenylalanine ammonia lyase was down-regulated in Otis plants under control conditions compared to Baronesse plants [-1.16 (RNA Seq) and −1.76 (qPCR)]. But it was up regulated in Otis under drought stress compared to Baronesse.

## Discussion

When compared with Otis, Baronesse was found to be more sensitive to drought as revealed by the biomass, leaf relative water content, proline accumulation, and the parameters associated with photosynthesis. Although significant decrease in the net photosynthetic rate (PN), stomatal conductance (gs), and the transpiration rate (E) was observed in both the barley genotypes under drought, but the degree of inhibition was less in the Otis compared to Baronesse. Indeed, this difference between the genotypes was supported by the RNA-Seq analysis that revealed a greater down regulation of several photosynthesis-related genes (genes for D1 protein (PsbA) and D2 protein (PsbD) in Baronesse compared to Otis. PSII (both D1 and D2 proteins are needed for assembly of a stable PSII complex) plays an important role in response to environmental stresses (Baker, 1991). In wheat genotypes, drought resulted in different degrees of repression of PsbA and PsbD genes, however, less repression of both genes (especially the PsbD gene) in the drought tolerant genotype (Liu et al., 2006).

Under well-watered conditions, Otis plants showed significantly less gs and E than Baronesse. This suggests that Otis might have lower stomatal density compared to Baronesse, which could be one player in drought tolerance of Otis. In line with this, the overexpression of epidermal patterning factor (EPF) (*HvEPF1*) in barley resulted in a significant reduction in stomatal density without adverse effects on the normal growth of the overexpression lines (Hughes et al., 2017). In addition, the overexpression lines showed less gs than the wild type under well-watered conditions. And under drought stress, they have higher water use efficiency and drought tolerance compared to the wild type. In drought tolerant rice and wheat genotypes, transpiration efficiency (TE) was enhanced by maximizing mesophyll conductance (gm) and minimizing stomatal conductance (gs) (Ouyang et al., 2017). Indeed, the drought tolerant rice and wheat showed low stomatal density, and thick mesophyll with thin cell walls.

### Correlations Between the Gene Expression Profiles of Proline and Glycine Betaine and Their Accumulation and Relative Water Content

Proline content was increased in both genotypes under drought, but the increase was significantly higher in Otis plants. Proline is an imino acid that acts as an osmoprotectant, a metal chelator, an antioxidative molecule, and a signaling molecule that enhances drought tolerance by maintaining the osmotic balance of the cells (Blum, 2009; Hayat et al., 2012). The LRWC of the two genotypes was significantly reduced, but drought treated Otis plants showed less reduction in their LRWC (Figure 2A). This response highlights one aspect of several mechanisms that could be used by Otis to tolerate drought better compared to Baronesse. High proline accumulation in the leaves of barley plants under severe osmotic stress leads to less membrane injury (Bandurska, 2001). In wild barley high proline accumulation in the leaves increased drought tolerance compared to cultivated barley (Bandurska and Stroihski, 2003). Indeed, in many major crop plants such as wheat, barley, and maize, osmotic adjustment was positively correlated with stress resistance (Blum, 2017). The expression of proline biosynthesis and turnover genes reflect this enhanced production of proline in both genotypes under drought. Pyrroline-5-carboxylate synthase (P5CS), pyrroline-5-carboxylate reductase, pyrroline-5-carboxylate dehydrogenase and Orn-δ-aminotransferase were all significantly induced in drought conditions. Only the proline turnover gene proline dehydrogenase expression was reduced under drought conditions. Although differences between the genotypes were not significant under reduced water conditions, the trend showed larger levels of expression in Otis (Supplementary Figure 1).

Glycine betaine (GB) is another important osmolyte and is known to accumulate in response to abiotic stresses in a variety of plant species (Ashraf and Foolad, 2007). GB is produced from choline via choline monooxygenase (CMO) and betaine aldehyde dehydrogenase (BADH). In barley, BADH1 and BADH2 genes were significantly induced under drought and salinity (Nakamura et al., 2001). Our results reveal that BADH (BART1_0-p47576, HORVU2Hr1G070680) was induced in both genotypes under drought. However, its expression was higher in Otis compared to Baronesse.

### Hormonal Profiles

Among the hormones, ABA is the most important hormone regarding its role in plant drought tolerance (Daszkowska-Golec, 2016; Sah et al., 2016; Vishwakarma et al., 2017). In this study, both genotypes accumulated ABA as well as several ABA-related metabolites under drought but the accumulation levels did not differ greatly between the genotypes. Likewise, the response of auxin levels was hardly differed between the genotypes under drought.

Cytokinins (CKs) and their metabolism is important in plants' adaptation to different abiotic stresses including drought (Ha et al., 2012; Pavlu et al., 2018). Drought stress caused a significant increase in the cytokinin, cZOG in Otis compared to Baronesse, suggesting a potential role for cZOG in the drought tolerance. Conjugation of O-glucose to cZ CKs (O-glucosylation) results in the formation of O-glucosides, and it is a reversible modification and the O-glucosides such as c-ZOG are storage forms of cZ CKs (Schäfer et al., 2015). A role for cis-Zeatin (cZ) CKs in plant growth and development has been reported (Kudo et al., 2012; Schäfer et al., 2015). It was also suggested that cZ CKs could be important for maintaining minimum CK activity for cell survival under stress conditions (Gajdošová et al., 2011).

With over 100 identified GAs, only a few are bioactive: GA1, GA3, and GA4 (Yamaguchi, 2008). The levels of GAs were found to be decreased under drought stress, and this could be associated with the retarded plant growth under stress (Nelissen et al., 2018). In small cereals (Tef and finger millet), the inhibition of GA biosynthesis resulted in an enhanced tolerance to osmotic stress (Plaza-Wüthrich et al., 2016). It was further reported that the reduction of GAs enhanced drought tolerance by osmotic adjustment and maintenance of leaf turgor of tomato (Omena-Garcia et al., 2019). Remarkably, GA20 levels were only increased in the drought-treated Otis. GA20 is an intermediate of GA1 and GA3, which is converted to GA1 by GA 3-oxidase (GA3ox) (Yamaguchi, 2008). This conversion was shown to be inhibited by heat, dehydration, and salinity (Colebrook et al., 2014). The increase in GA20 in the drought-treated Otis suggests a less conversion of this gibberellin to the bioactive forms of GA.

For hormonal profiling, samples of barley leaves were taken at the initial wilting stage of drought (5 days of drought). At this stage, no significant changes were observed for shoot length or biomass in the two genotypes under drought stress compared to the control. This might explain the observed small number of differences regarding hormonal profiles under drought in the genotypes.

### General Transcriptional Responses Greatly Differed Between the Genotypes

The drought sensitive genotype Baronesse showed higher number of differentially expressed genes (DEGs) under drought (1,023, and 786 up and down-regulated, respectively), compared to Otis (460, and 314 up and down-regulated genes, respectively), in the drought-tolerant Otis indicating that the transcriptional changes were far greater in sensitive genotype. Indeed, previous studies have reported a greater number of stress-regulated genes in the sensitive genotypes compared to the tolerant genotypes subjected to stress treatments (Silveira et al., 2015; Cantalapiedra et al., 2017; Janiak et al., 2019; Ereful et al., 2020).

### The Shared Responses Between Otis and Baronesse as Revealed by the Differentially Expressed Genes

In this study, several differentially regulated genes (signaling genes (kinases and phosphatases), transcription factors, chaperones, annexins, and aquaporins) that showed almost similar level of regulation in both the genotypes under drought have been identified and these could be important for maintaining cellular homeostasis under stress.

Chaperones have been shown to stabilize membranes and proteins by assisting with folding, association, translocation, and degradation of proteins under stress (Priya et al., 2019). The chaperone gene BART1_0-p34164 (HORVU5Hr1G027910) was up regulated in the drought-treated Otis and Baronesse (Log2 FC is 1.55 and 1.44, respectively). The ortholog of this gene in Arabidopsis was shown to be induced under drought stress (Gupta and Senthil-Kuma, 2017).

Aquaporins (AQPs) are pore forming proteins belonging to the major intrinsic proteins (MIP) superfamily which transport water and other small neutral compounds across the membrane. The upregulation of AQPs in response to abiotic stresses is known in plants (Quigley et al., 2001; Scharwies, 2017; Kapilan et al., 2018). The aquaporin, BART1_0-p07678, HORVU2Hr1G010990 was significantly induced in both the genotypes under drought stress. The rice ortholog (OsPIP2.6) of this gene (LOC_Os04g16450), has shown to be induced in the drought tolerant parent and the inbred lines but repressed in the drought sensitive parent (Baghyalakshmi et al., 2020).

Annexins are a diverse, multigene family of calcium-dependent, membrane-binding proteins that serve as targets for Ca2+ in most eukaryotic cells (Clark et al., 2001). An annexin gene (BART1_0-p51213, HORVU7Hr1G037080) was one among the commonly up regulated genes in Otis and Baronesse under drought stress (Log2 FC is 3.08 and 3.57, respectively). The orthologous gene in rice (LOC_Os06g11800) was shown to be upregulated under drought stress (Gorantla et al., 2005). The Arabidopsis ortholog of Annexin 1 (AT1G35720) was also induced by drought and its overexpression confers enhanced drought tolerance (Konopka-Postupolska et al., 2009). A possible mode of Annexin 1 in drought tolerance include the alleviation of the oxidation of the membrane's lipids and proteins (Jami et al., 2008).

### A Gene for Wax Biosynthesis was Uniquely and Highly Induced in Otis Genotype Under Drought

*ECERIFERUM1* (*CER1*) gene [CER1 from fatty acid hydrolase superfamily (BART1_0-p02677)] involved in wax biosynthesis was highly upregulated in Otis under drought. The Arabidopsis and rice orthologs of this gene are *CER1* (AT1G02205) and WAX2 (LOC_Os10g33250), respectively. In drought treated Arabidopsis plants, the expression of *CER1* gene was up regulated, along with a significant increase in the very long chain (VLC) alkanes in the cuticle (Kosma et al., 2009). The Arabidopsis *CER1* gene codes for an important enzyme involved in the biosynthesis of VLC alkanes of the cuticle (Bourdenx et al., 2011). The cuticle is a hydrocarbon epidermal extension, that acts as a protective barrier against water loss under drought stress. Indeed, the overexpression of *CER1* in Arabidopsis conferred drought tolerance due to reduced water loss (Bourdenx et al., 2011). In Brachypodium, eight homologs of *CER1* were identified (Wu et al., 2019). *BdCER1-8* was highly expressed in the leaves, and it was significantly induced by drought and osmotic stress. Moreover, *BdCER1-8* rescued the biosynthesis of the VLC alkanes in cer1 Arabidopsis mutant (Wu et al., 2009). The overexpression of the transcription factor *TaSHN1* in wheat resulted in reduced stomatal density and leaf water loss, and thereby improved drought tolerance of the transgenic lines (Bi et al., 2018). In addition, the analysis of the cuticle composition of *TaSHN1*overexpression lines revealed a significant increase in the alkanes under control and drought conditions. The overexpression of *TaSHN1* also resulted in more than 10-fold upregulation of the cuticle biosynthetic genes such as: *ATT1/CYP86, CER4-6, KCS1*, and *LACS3*. This indicates that one mechanism of the improved drought tolerance in the *TaSHN1* overexpression lines is through changes in the cuticle composition both at the molecular and biochemical levels. The induction of CER1 in the drought-treated Otis is one of the few major differences of supporting drought tolerant nature of Otis compared to Baronesse. This might explain the less reduction in the leaf relative water content in Otis under drought stress. Indeed, a detailed analysis of the cuticle composition of Otis and Baronesse under drought and control conditions will shed more light on drought tolerance in barley.

### A Beta-Expansin Gene was Uniquely and Highly Induced in Otis Genotype Under Drought

Expansin gene family is one group of cell wall modifying genes (Cosgrove, 2000). Expansin genes are important players in cell growth through loosening of the cell wall (Cosgrove, 2015). Phylogenetically, expansin genes are divided into two major families: EXPA (α-expansins) and EXPB (β-expansins) (Cosgrove, 2015). In barley, a total of 34 expansin genes (14 EXPA, 17 EXPB, and 3 EXPLA) were identified (Lombardi, 2012). The expression of the barley expansin genes showed specific expression profile for each tissue, organ, and developmental stage. Previous studies showed the involvement of expansin genes in many growth and developmental processes (Choi et al., 2003; Marowa et al., 2016). Moreover, expansin genes were found to be differentially expressed under different abiotic stresses (Wu et al., 2001; Bray, 2004; Harb et al., 2010; Marowa et al., 2016). The overexpression of TaEXPA2 in tobacco and wheat enhanced drought tolerance in the transgenic plants (Chen et al., 2016; Yang et al., 2020). Moreover, improved tolerance to salinity and drought was also shown in tobacco plants overexpressing the tobacco EXPA4 (Chen et al., 2018). In barley, HvEXPB7 improved the growth of barley root hairs under drought in the drought tolerant Tibetan wild barley genotype (He et al., 2015). The beta expansin gene (BART1_0-p22302) was highly induced in the drought stressed Otis (Log2 FC is 6). The Arabidopsis and rice orthologs of this gene are AT1G65680 (AtEXPB2), and LOC_Os03g01270 (OsEXPB7). The rice gene was significantly repressed under dehydration conditions (Zhou et al., 2007; Ray et al., 2011; Shaik and Ramakrishna, 2012). In these studies, the changes in gene expression were tested in one genotype of rice under dehydration stress, which is considered as a shock stress to plants. Whereas, in this study, two barley genotypes with contrasting drought tolerance were exposed to progressive drought for 5 days. Indeed, a low correlation between gene expression under dehydration shock and that under progressive (gradual) drought was shown in barley (Talamé et al., 2007).

### An Armadillo (ARM) Repeat Gene is Highly Upregulated in Otis Genotype Under Drought

Armadillo (ARM) repeat gene family has the ARM repeat domain, which is composed of one short and two relatively longer α-helices (Mudgil et al., 2004; Sharma and Pandey, 2016). It includes members of diverse functions such as: signal transduction, nuclear transport, cell adhesion, and protein degradation (Sharma et al., 2014). The most common protein arrangement of ARM family is U-box/ARM (PUB/ARM), which suggests a role in protein ubiquitination (Sharma and Pandey, 2016). This will result in higher plasticity in response to the changing environments. The Armadillo gene was among the drought responsive genes in the drought tolerant potato genotype (Pieczynski et al., 2018). In rice plants, 36 OsARM genes were differentially expressed under different abiotic stresses (drought, salt, and cold) (26 up-regulated and 10 down-regulated) (Sharma et al., 2014). Out of the 26 up-regulated genes 7 were uniquely drought induced genes, and 4 out of 10 were uniquely drought repressed genes. In barley, 5 PUB/ARM genes [class II U-box E3 ubiquitin ligases (HvPUB7, HvPUB9, HvPUB15, HvPUB16, HvPUB21, and HvPUB22)] were significantly induced, and one gene (HvPUB18) was repressed under dehydration stress (Ryu et al., 2019). The ARM repeat gene (BART1_0-p34106) was highly induced in Otis drought stressed plants (Log2 FC 5.88). This gene is not a member of the U-box E3 ubiquitin ligase family in barley (Ryu et al., 2019). This suggests that it might function in drought tolerance of Otis via mechanism (s) other than protein ubiquitination and degradation.

### Kinases are Predominantly Down Regulated in Baronesse Plants Under Drought

Genes encoding protein kinases (Pks) were overrepresented among the down regulated genes in drought-treated Baronesse. Most of these kinases are receptor like kinase-Pelle (RLK-Pelle). Receptor like kinase-Pelle is the largest gene family in Arabidopsis and rice, which are responsive to different abiotic and biotic stresses (Lehti-Shiu et al., 2009). In rice, receptor-like cytoplasmic kinase GROWTH UNDER DROUGHT KINASE (GUDK) was shown to improve drought tolerance through the activation of the transcription factor APETALA2/ETHYLENE RESPONSE FACTOR OsAP37 (Ramegowda et al., 2014). The overexpression of poplar leucine-rich repeat (LRR) receptor-like kinase in Arabidopsis enhanced water use efficiency (Xing et al., 2011). The general down regulation of many kinases in Baronesse relative to Otis could be one of the factors associated with drought sensitivity.

### Transcription Factors

Transcription factors (TFs) are regulatory proteins that play an important role in almost all plant processes including adaptation to biotic and abiotic stresses (Nakashima et al., 2014; Joshi et al., 2016). TF genes such as AP2/EREBP, bZIP, MYB/MYC, NAC, WRKY have been implicated in drought stress responses (Gahluat et al., 2016). The number of DEGs encoding TFs were more in the drought-stressed Baronesse than in Otis.

NAC genes are plant-specific transcription factors involved in growth and development and stress responses. Overexpression of several NAC genes from Arabidopsis, rice, and soybean increased the tolerance of transgenic plants to abiotic stresses including drought (Nakashima et al., 2007; Hao et al., 2011; Wang and Dane, 2013; Shim et al., 2018). In drought stressed Otis, two NAC genes were up regulated, while one NAC gene was down regulated. The overexpression of wheat NAC TF improved tolerance to drought and salt stress in Arabidopsis (Huang et al., 2015).

One of the induced NAC genes in Otis genotype is BART1_0-p58823 (HORVU0Hr1G017490), which has Log2FC of 5.33. The rice ortholog of this gene is LOC_Os02g56600, which was induced in the salt-tolerant rice genotype but repressed in the salt-sensitive genotype (García-Morales et al., 2014). The other NAC gene that was induced specifically in the Otis genotype is BART1_0-p12809 (HORVU2Hr1G080460), which has Log2FC of 2.95. The ortholog of this gene in rice is LOC_Os04g38720 (OsNAC2), which was also induced by salt stress (Narsai et al., 2013). The one NAC gene that was uniquely repressed (Log2FC of−2.09) in Otis under drought is BART1_0-p22840 (HORVU3Hr1G080100). The rice ortholog of this gene is OsNAC4 (LOC_Os01g60020), which was induced in the dehydrated wild rice (*Oryza rufipogon*) (Zhang et al., 2017). Another study showed this gene was highly induced in the drought tolerant rice genotype than the sensitive genotype after 3 hours of dehydration (Borah et al., 2017). In the two previous studies, rice plants were exposed to a dehydration shock rather than a progressive drought treatment.

The rice ortholog of BART1_0-p13794 gene is OsHOX22 (LOC_Os04g45810), which was induced by desiccation, salinity, cold, and osmotic stresses (Bhattacharjee et al., 2016). Moreover, it was among the drought expressed genes in rice in the co-expression analysis of different transcriptome datasets (Lv et al., 2019). The expression level of this gene greatly differed between Otis and Baronesse (3.17 and 1.75 Log2FC, Otis and Baronesse, respectively). The higher induction of this gene in Otis might be important for drought tolerance in this genotype.

Tryptophan-arginine-tyrosine (WRKY) proteins are one of the largest families of transcription factors specific to plants (Zhang and Wang, 2005). Many WRKY genes have been shown to be induced by abiotic stresses including drought (Chen et al., 2011). Their overexpression resulted in improved drought tolerance in different plant species (Wu et al., 2009; Cai et al., 2014; Xu et al., 2014; Ding et al., 2016). In this study, 4 out of the 21 drought-repressed TFs in the Baronesse plants were WRKY TFs with Log2 FC ≤ −2. These WRKY genes are: BART1_0-p09203 (HORVU2Hr1G029450), BART1_0-p23505 (HORVU3Hr1G088200), BART1_0-p01968 (HORVU1Hr1G027700), and BART1_0-p21247 (HORVU3Hr1G059210) with Log2FC−3.22,−2.31,−2.82, and−2.95, respectively. The rice orthologs of these genes are: OsWRKY69 (LOC_Os08g29660), OsWRKY24 (LOC_Os01g61080), OsWRKY67 (LOC_Os05g09020), and OsWRKY15 (LOC_Os01g46800). OsWRKY69 was up regulated in the leaves and root of drought tolerant rice genotype (Silveira et al., 2015; Baldoni et al., 2016). Whereas, OsWRKY24, OsWRKY67, and OsWRKY15 were found to play a role in tolerance of phosphorus deficiency, bacterial resistance, and cold tolerance, respectively (Yang et al., 2015; Deng et al., 2018; Liu Q. et al., 2018).

### Alternative Splicing

Alternative splicing (AS) is an important posttranscriptional mechanism in which different combinations of exons of a primary transcript are joined to produce diverse messenger RNA (mRNA) isoforms. Interestingly, the abiotic stresses were shown to alter the AS pattern in plants (Reddy et al., 2013; Laloum et al., 2018). In this study only a relatively small number of AS genes (37 genes in Otis and 61 genes in Baronesse) were identified in barley genotypes exposed to drought (Table 1). It was reported previously that the DAS events were relatively smaller under drought compared to other abiotic stresses. For example, in wheat, only 200 genes undergoing DAS under drought while this number is rather high (3,576 genes) under heat stress (Liu Z. et al., 2018). In Cassava, only 1,025 genes showed DAS in response to drought stress compared to 3,292 genes in response to cold stress (Li et al., 2020). In maize, 1,045 and 985 genes showed DAS under heat and cold stresses, respectively, while only 281 and 204 genes showed DAS during drought stress in ovary and leaf, respectively, and only 14 of these DAS genes were common to both tissues indicating a tissue-specific differences (Mei et al., 2017). These studies suggest that alternative splicing is less frequently used under drought compared to other abiotic stresses. The results here also show genotype-specific differences in DAS responses under drought.

Interestingly, the proteins involved in splicing were also modulated by stress conditions (Ali and Reddy, 2008). In the present study, we found that the levels of two splicing factors (RS31 and SCL30) were significantly upregulated in drought-stressed Otis and Baronesse (Figure 7A). Arabidopsis orthologs of these genes have been found to regulate plant splice site selection and it is possible that changes in their expression will also lead to DAS in barley (Lopato et al., 1996; Yan et al., 2017). Remarkably, most DAS events were genotype-specific in this study indicating differences in AS between the two barley genotypes. Some of these transcript changes led to a switch from one major abundant isoform to an alternative transcript, which became the abundant transcript isoform under drought stress (Figure 7B; Supplementary Figure 2). Such large changes in AS transcript abundances have been described previously in human cancers and were considered as post-transcriptional biological markers of the condition (Climente-González et al., 2017). We found drought induced AS events affecting exon skipping and changes in the selection of alternative 5' and 3' splice sites. But many stress changes led to transcripts that show intron retention. In some cases, the switched transcript under drought led to a transcript with an intron retention (Figure 7B is one example). Intron retentions alters the length of the 3'UTR and may affect transcript stability or transport from the nucleus (Kalyna et al., 2012; Göhring et al., 2014). Overall, DAS affects a small number of genes in the two genotypes under drought stress but alters the abundance of the gene transcripts in a highly significant manner and it remains to be determined the importance of such changes.

## Conclusions

Drought tolerance is a complex process involving several thousands of genes associated with various biochemical and physiological processes. In this study, two barley genotypes differing in their drought tolerance (Otis and Baronesse) were compared for their molecular, hormonal, and physiological differences under drought. Otis had better photosynthetic capacity under drought compared to Baronesse, which could be attributed to the differences in gene expression (D1 and D2) associated with PSII stability. The hormone analysis revealed that both genotypes showed significant induction of ABA under stress conditions. Similarly, at the molecular level, many stress responsive genes such as chaperones, aquaporins, and annexins were found to be regulated at similar levels in both genotypes under drought stress. However, a few genes such as BADH and homeobox TF were highly induced in Otis compared to Baronesse. Importantly, many potential drought tolerance genes such as wax biosynthesis gene (CER1), and two NAC TFs were uniquely induced in Otis under drought stress. On the other hand, genes for WRKY TFs, and PKs were highly down-regulated in the drought-stressed Baronesse. Taken together, the overall transcriptional responses were low in drought-tolerant Otis but the genes that could confer drought tolerance were either specifically induced or greatly upregulated in the tolerant genotype and these differences could be important for drought tolerance in barley.

## Data Availability Statement

The datasets generated for this study can be found in online repositories. The names of the repository/repositories and accession number(s) can be found at: European Nucleotide Archive (ENA) at EMBL-EBI under accession number PRJEB40905.

## Author Contributions

AH and RS conceived the idea. AH conducted the drought treatments and harvested samples as well as estimation of biochemical parameters. CS and WG performed the bioinformatics analysis of RNA-Seq datasets. VGK assisted with measurements of photosynthesis-related parameters. GG performed qPCR analysis. AH prepared the manuscript with contributions from WG and CS. RS, CS, and AH refined and finalized the draft. All authors reviewed and approved the final manuscript.

## Conflict of Interest

The authors declare that the research was conducted in the absence of any commercial or financial relationships that could be construed as a potential conflict of interest.

## References

[B1] Ali G. S. Reddy A. S. N. (2008). Regulation of alternative splicing of pre-mRNAs by stresses, in Nuclear pre-mRNA Processing in Plants. Current Topics in Microbiology and Immunology, eds Reddy A. S. N. Golovkin M. (Berlin: Springer Berlin Heidelberg), 257–275. 10.1007/978-3-540-76776-3_14 18630757

[B2] Ashraf M. Foolad M. R. (2007). Roles of glycine betaine and proline in improving plant abiotic stress resistance. Environ. Exp. Bot. 59, 206–216. 10.1016/j.envexpbot.2005.12.006

[B3] Baghyalakshmi K. Ramchander S. Raveendran M. Jeyaprakash P. (2020). Unravelling of osmotic genes involved in, drought tolerance in Backcross inbred lines of rice (*Oryza sativa L*.) cultivars. Res. J. Biotech. 15, 52–60. Available online at: https://www.researchgate.net/profile/Ramchander_Selvaraj2/publication/342623213_Unravelling_of_Osmotic_genes_involved_in_Drought_tolerance_in_Backcross_inbred_lines_of_rice_Oryzasativa_L_cultivars/links/5efd58a7458515505084919d/Unravelling-of-Osmotic-genes-involved-in-Drought-tolerance-in-Backcross-inbred-lines-of-rice-Oryzasativa-L-cultivars.pdf

[B4] Baker N. (1991). A possible role for photosystem II in environmental perturbations of photosynthesis. Physiol. Plant. 81, 563–570. 10.1111/j.1399-3054.1991.tb05101.x

[B5] Baldoni E. Bagnaresi P. Locatelli F. Mattana M. Genga A. (2016). Comparative leaf and root transcriptomic analysis of two rice Japonica cultivars reveals major differences in the root early response to osmotic stress. Rice 9, 25–45. 10.1186/s12284-016-0098-1 27216147 PMC4877341

[B6] Bandurska H. (2001). Does proline accumulated in leaves of water deficit stressed barley plants confine cell membrane injuries? II. Proline accumulation during hardening and its involvement in reducing membrane injuries in leaves subjected to severe osmotic stress. Acta Physiol. Plant. 23, 483–490. 10.1007/s11738-001-0059-0

[B7] Bandurska H. Stroihski A. (2003). ABA and proline accumulation in leaves and roots of wild (*Hordeum spontaneum*) and cultivated (*Hordeum vulgate 'Maresi'*) barley genotypes under water deficit conditions. Acta Physiol. Plant 25, 55–61. 10.1007/s11738-003-0036-x

[B8] Bartels D. Sunkar R. (2005). Drought and salt tolerance in plants. Crit. Rev. Plant Sci. 24, 23–58. 10.1080/07352680590910410

[B9] Benjamini Y. Yekutieli D. (2001). The control of the false discovery rate in multiple testing under dependency. Ann. Statist. 29, 1165–1188. 10.1214/aos/1013699998

[B10] Bhattacharjee A. Khurana J. P. Jain M. (2016). Characterization of rice homeobox genes, OsHOX22 and OsHOX24, and over-expression of OsHOX24 in transgenic Arabidopsis suggest their role in abiotic stress response. Front. Plant Sci. 7:627. 10.3389/fpls.2016.00627 27242831 PMC4862318

[B11] Bi H. Shi J. Kovalchuk N. Luang S. Bazanova N. Chirkova L.. (2018). Overexpression of the *TaSHN1* transcription factor in bread wheat leads to leaf surface modifications, improved drought tolerance, and no yield penalty under controlled growth conditions. Plant Cell Environ. 41, 2549–2566. 10.1111/pce.13339 29761511

[B12] Bielach A. Hrtyan M. Tognetti V. (2017). Plants under stress: involvement of auxin and cytokinin. Int. J. Mol. Sci. 18, 1427–1456. 10.3390/ijms18071427 28677656 PMC5535918

[B13] Blum A. (2009). Effective use of water (EUW) and not water-use efficiency (WUE) is the target of crop yield improvement under drought stress. Field Crops Res. 112, 119–123. 10.1016/j.fcr.2009.03.009

[B14] Blum A. (2017). Osmotic adjustment is a prime drought stress adaptive engine in support of plant production. Plant Cell Environ. 40, 4–10. 10.1111/pce.12800 27417527

[B15] Borah P. Sharma E. Kaur A. Chandel G. Mohapatra T. Kapoor S. (2017). Analysis of drought-responsive signalling network in two contrasting rice cultivars using transcriptome-based approach. Sci. Rep. 7:42131 10.1038/srep42131 28181537 PMC5299611

[B16] Bourdenx B. Bernard A. Domergue F. Pascal S. Le'ger A. Roby D.. (2011). Overexpression of Arabidopsis ECERIFERUM1 promotes wax very-long-chain alkane biosynthesis and influences plant response to biotic and abiotic stresses. Plant Physiol. 156, 29–45. 10.1104/pp.111.172320 21386033 PMC3091054

[B17] Bray E. A. (2004). Genes commonly regulated by water-deficit stress in *Arabidopsis thaliana*. J. Exp. Bot. 55, 2331–2341. 10.1093/jxb/erh270 15448178

[B18] Bullard J. H. Purdom E. Hansen K. D. Dudoit S. (2010). Evaluation of statistical methods for normalization and differential expression in mRNA-Seq experiments. BMC Bioinformatics 11, 94–107. 10.1186/1471-2105-11-94 20167110 PMC2838869

[B19] Burton R. Shirley N. King B. Harvey A. Fincher G. (2004). The CesA. quantitative analysis of transcripts reveals two groups of co-expressed genes. Plant Physiol. 134, 224–236. 10.1104/pp.103.032904 14701917 PMC316302

[B20] Cafiero C. Viviani S. Nord M. (2018). Food security measurement in a global context: the food insecurity experience scale. Measurement 116, 146–152. 10.1016/j.measurement.2017.10.065

[B21] Cai R. Zhao Y. Wang Y. Lin Y. Peng X. Li Q. (2014). Overexpression of a maize WRKY58 gene enhances drought and salt tolerance in transgenic rice. Plant Cell Tissue Organ Cult. 119, 565–577. 10.1007/s11240-014-0556-7

[B22] Calixto C. P. G. Guo W. James A. B. Tzioutziou N. A. Entizne J. C. Panter P. E.. (2018). Rapid and dynamic alternative splicing impacts the arabidopsis cold response transcriptome. Plant Cell 30, 1424–1444. 10.1105/tpc.18.00177 29764987 PMC6096597

[B23] Cantalapiedra C. P. García-Pereira M. J. Gracia M. P. Igartua E. Casas A. M. Contreras-Moreira B. (2017). Large differences in gene expression responses to drought and heat stress between elite barley cultivar Scarlett and a Spanish landrace. Front. Plant Sci. 8:647. 10.3389/fpls.2017.00647 28507554 PMC5410667

[B24] Carillo P. Gibon Y. (2011). Protocol: Extraction and Determination of Proline. Available online at: http://prometheuswiki.org/tiki-index.php?page=Extraction+and+determination+of+proline (accessed December 6, 2019).

[B25] Chen L. Song Y. Li S. Zhang L. Zou C. Yu D. (2011). The role of WRKY transcription factors in plant abiotic stresses. Biochim. Biophys. Acta. 1819, 120–128. 10.1016/j.bbagrm.2011.09.002 21964328

[B26] Chen L. J. Zou W. S. Fei C. Y. Wu G. Li X. Y. Lin H. H.. (2018). α-expansin *EXPA4* positively regulates abiotic stress tolerance but negatively regulates pathogen resistance in *Nicotiana tabacum*. Plant Cell Physiol. 59, 2317–2330. 10.1093/pcp/pcy155 30124953

[B27] Chen Y. Han Y. Zhang M. Zhou S. Kong X. Wang W. (2016). Overexpression of the wheat expansin gene *TaEXPA2* improved seed production and drought tolerance in transgenic tobacco plants. PLoS ONE 11:e0153494. 10.1371/journal.pone.0153494 27073898 PMC4830583

[B28] Choi D. Lee Y. Cho H. T. Kende H. (2003). Regulation of expansin gene expression affects growth and development in transgenic rice plants. Plant Cell 15, 1386–1398. 10.1105/tpc.011965 12782731 PMC156374

[B29] Clark G. B. Sessions A. Eastburn D. J. Roux S. J. (2001). Differential expression of members of the annexin multigene family in *Arabidopsis*. Plant Physiol. 126, 1072–1084. 10.1104/pp.126.3.1072 11457958 PMC116464

[B30] Climente-González H. Porta-Pardo E. Godzik A. Eyras E. (2017). The functional impact of alternative splicing in cancer. Cell Rep. 20, 2215–2226. 10.1016/j.celrep.2017.08.012 28854369

[B31] Colebrook E. Thomas S. Philips A. Hedden P. (2014). The role of gibberellin signalling in plant responses to abiotic stress. J. Exp. Bot. 217, 67–75. 10.1242/jeb.089938 24353205

[B32] Cosgrove D. (2000). Expansive growth of plant cell walls. Plant Physiol. Biochem. 38, 109−124. 10.1016/S0981-9428(00)00164-9 11543185

[B33] Cosgrove D. (2015). Plant expansins: diversity and interactions with plant cell walls. Curr. Opin. Plant Biol. 25, 162–172. 10.1016/j.pbi.2015.05.014 26057089 PMC4532548

[B34] Daszkowska-Golec A. (2016). The role of abscisic acid in drought stress: how ABA helps plants to cope with drought stress, in Drought Stress Tolerance in Plants, Vol 2, eds Hossain M. Wani S. Bhattacharjee S. Burritt D. Tran L. S. (Cham: Springer), 123–151. 10.1007/978-3-319-32423-4_5

[B35] Dawson I. Russell J. Powell W. Steffenson B. Thomas W. Waugh R. (2015). Barley: a translational model for adaptation to climate change. New Phytol. 206, 913–931. 10.1111/nph.13266 25605349

[B36] de Mezer M. Turska-Taraska A. Kaczmarek Z. Glowacka K. Swarcewicz B. Rorat T. (2014). Differential physiological and molecular response of barley genotypes to water deficit. Plant Physiol. Biochem. 80, 234–248. 10.1016/j.plaphy.2014.03.025 24811679

[B37] Deng Q. Luo X. Chen Y. Zhou Y. Zhou Y. Zhang F. (2018). Transcriptome analysis of phosphorus stress responsiveness in the seedlings of Dongxiang wild rice (*Oryza rufpogon Grif*.). Biol. Res. 51, 7–19. 10.1186/s40659-018-0155-x 29544529 PMC5853122

[B38] Diab A. Teulat-Merah B. This D. Ozturk N. Z. Benscher D. Sorrells M. E. (2004). Identification of drought-inducible genes and differentially expressed sequence tags in barley. Theor. Appl. Genet. 109, 1417–1425. 10.1007/s00122-004-1755-0 15517148

[B39] Ding W. W. Fang W. B. Shi S. Y. Zhao Y. Y. Li X. J. Xiao K. (2016). Wheat WRKY type transcription factor gene TaWRKY1 is essential in mediating drought tolerance associated with an ABA-dependent pathway. Plant Mol. Biol. Rep. 34, 1111–1126. 10.1007/s11105-016-0991-1

[B40] Duque P. (2011). A role for SR proteins in plant stress responses. Plant Signal. Behav. 6, 49–54. 10.4161/psb.6.1.14063 21258207 PMC3122005

[B41] Ereful N. C. Liu L. Greenland A. Powell W. Mackay I. Leung H. (2020). RNA-seq reveals differentially expressed genes between two indica inbred rice genotypes associated with drought-yield QTLs. Agron 10, 621–640. 10.3390/agronomy10050621

[B42] Gahluat V. Jaiswal V. Kumar A. Gupta P. K. (2016). Transcription factors involved in drought tolerance and their possible role in developing drought tolerant cultivars with emphasis on wheat (*Triticum aestivum* L.). Theor. Appl. Genet. 129, 2019–2042. 10.1007/s00122-016-2794-z 27738714

[B43] Gajdošová S. Spíchal L. Kamínek M. Hoyerová K. Novák O. Dobrev P.. (2011). Distribution, biological activities, metabolism, and the conceivable function of cis-zeatin-type cytokinins in plants. J. Exp. Bot. 62, 2827–2840. 10.1093/jxb/erq457 21282330

[B44] García-Morales S. Gómez-Merino F. Trejo-Téllez L. (2014). NAC transcription factor expression, amino acid *concentration* and growth of elite rice cultivars upon salt stress. Acta Physiol. Plant. 36, 1927–1936. 10.1007/s11738-014-1569-x

[B45] Giraldo P. Benavente E. Manzano-Agugliaro F. Gimenez E. (2019). Worldwide research trends on wheat and barley: a bibliometric comparative analysis. Agron 9, 352–370. 10.3390/agronomy9070352

[B46] Göhring J. Jacak J. Barta A. (2014). Imaging of endogenous messenger RNA splice variants in living cells reveals nuclear retention of transcripts inaccessible to nonsense-mediated decay in Arabidopsis. Plant Cell 26, 754–764. 10.1105/tpc.113.118075 24532591 PMC3967038

[B47] Gorantla M. Babu P. R. Lachagari V. B. R. Feltus F. A. Paterson A. H. Reddy A. R. (2005). Functional genomics of drought-stress response in rice: transcript mapping of annotated unigenes of an indica rice (*Oryza sativa L*. cv. Nagina 22). Curr. Sci. 89, 496–514. Available online at: http://www.iisc.ernet.in/currsci/aug102005/496.pdf

[B48] Guo P. Baum M. Grando S. Ceccarelli S. Bai G. Li R.. (2009). Differentially expressed genes between drought-tolerant and drought-sensitive barley genotypes in response to drought stress during the reproductive stage. J. Exp. Bot. 60, 3531–3544. 10.1093/jxb/erp194 19561048 PMC2724701

[B49] Guo Y. Ping W. Chen J. Zhu L. Zhao Y. Guo J.. (2019). Meta-analysis of the effects of overexpression of WRKY transcription factors on plant responses to drought stress. BMC Genetics 20, 63–77. 10.1186/s12863-019-0766-4 31349781 PMC6660937

[B50] Gupta A. Senthil-Kuma M. (2017). Transcriptome changes in *Arabidopsis thaliana* infected with *Pseudomonas syringae* during drought recovery. Sci. Rep. 7:9124. 10.1038/s41598-017-09135-y 28831155 PMC5567376

[B51] Ha S. Vankova R. KazukoYamaguchi-Shinozaki K. Shinozaki K. Tran L. S. (2012). Cytokinins: metabolism and function in plant adaptation to environmental stresses. Trends Plant Sci. 17, 172–179. 10.1016/j.tplants.2011.12.005 22236698

[B52] Hao Y. J. Wei W. Song Q. X. Chen H. W. Zhang Y. Q. Wang F.. (2011). Soybean NAC transcription factors promote abiotic stress tolerance and lateral root formation in transgenic plants. Plant J. 68, 302–313. 10.1111/j.1365-313X.2011.04687.x 21707801

[B53] Harb A. Krishnan A. Ambavaram M. M. Pereira A. (2010). Molecular and physiological analysis of drought stress in *Arabidopsis* reveals early responses leading to acclimation in plant growth. Plant Physiol. 54, 1254–1271. 10.1104/pp.110.161752 20807999 PMC2971604

[B54] Harb A. Samarah N. (2015). Physiological and molecular responses to controlled severe drought in two barley (*Hordeum Vulgare* L.) genotypes. J. Crop Improv. 29, 82–94. 10.1080/15427528.2014.976802

[B55] Hasanuzzaman M. Shabala L. Brodribb T. Zhou M. Shabala S. (2019). Understanding physiological and morphological traits contributing to drought tolerance in barley. J. Agron. Crop Sci. 205, 129–140. 10.1111/jac.12307

[B56] Hayat S. Hayat Q. Alyemeni M. N. Wani A. Pichtel J. Ahmad A. (2012). Role of proline under changing environments. Plant Signal. Behav. 7, 1456–1466. 10.4161/psb.21949 22951402 PMC3548871

[B57] He X. Zeng J. Cao F. Ahmed I. M. Zhang G. Vincze E.. (2015). HvEXPB7, a novel β-expansin gene revealed by the root hair transcriptome of Tibetan wild barley, improves root hair growth under drought stress. J. Exp. Bot. 66, 7405–7749. 10.1093/jxb/erv436 26417018 PMC4765802

[B58] Heath R. Packer L. (1968). Photoperoxidation in isolated chloroplasts. I. Kinetics and stoichiometry of fatty acid peroxidation. Arch. Biochem. Biophys. 125, 189–198. 10.1016/0003-9861(68)90654-1 5655425

[B59] Hiei Y. Ishida Y. Komari T. (2014). Progress of cereal transformation technology mediated by *Agrobacterium tumefaciens*. Front. Plant Sci. 5, 628–640. 10.3389/fpls.2014.00628 25426132 PMC4224067

[B60] Huang Q. Wang Y. Li B. Chang J. Chen M. Li K. (2015). TaNAC29, a NAC transcription factor from wheat, enhances salt and drought tolerance in transgenic Arabidopsis. BMC Plant Biol. 15, 268–283. 10.1186/s12870-015-0644-9 26536863 PMC4632686

[B61] Hübner S. Korol A. B. Schmid K. J. (2015). RNA-Seq analysis identifies genes associated with differential reproductive success under drought-stress in accessions of wild barley *Hordeum spontaneum*. BMC Plant Biol. 5, 134–148. 10.1186/s12870-015-0528-z PMC4459662 26055625

[B62] Hughes J. Hepworth C. Dutton C. Dunn J. Hunt L. Stephens J.. (2017). Reducing stomatal density in barley improves drought tolerance without impacting on yield. Plant Physiol. 174, 776–787. 10.1104/pp.16.01844 28461401 PMC5462017

[B63] Jami S. K. Clark G. B. Turlapati S. A. Handley C. A. Roux S. J. Kirti P. B. (2008). Ectopic expression of an annexin from Brassica juncea confers tolerance to abiotic and biotic stress treatments in transgenic tobacco. Plant Physiol. Biochem. 46, 1019–1030. 10.1016/j.plaphy.2008.07.006 18768323

[B64] Janiak A. Kwasniewski M. Sowa M. Kuczyńska A. Mikołajczak K. Ogrodowicz P.. (2019). Insights into barley root transcriptome under mild drought stress with an emphasis on gene expression regulatory mechanisms. Int. J. Mol. Sci. 20, 6139–6164. 10.3390/ijms20246139 31817496 PMC6940957

[B65] Joshi R. Wani S. H. Singh B. Bohra A. Dar Z. A. Lone A. A.. (2016). Transcription factors and plants response to drought stress: current understanding and future directions. Front. Plant Sci. 7, 1029–1044. 10.3389/fpls.2016.01029 27471513 PMC4943945

[B66] Kalyna M. Simpson C. G. Syed N. H. Lewandowska D. Marquez Y. Kusenda B.. (2012). Alternative splicing and nonsense-mediated decay modulate expression of important regulatory genes in *Arabidopsis*. Nucleic Acids Res. 40, 2454–2469. 10.1093/nar/gkr932 22127866 PMC3315328

[B67] Kapilan R. Vaziri M. Zwiazek J. J. (2018). Regulation of aquaporins in plants under stress. Biol. Res. 51, 4–16. 10.1186/s40659-018-0152-0 29338771 PMC5769316

[B68] Kim W. Iizumi T. Nishimori M. (2019). Global patterns of crop production losses associated with droughts from 1983 to 2009. J. Appl. Meteorol. Climatol. 58, 1233–1244. 10.1175/JAMC-D-18-0174.1

[B69] Konopka-Postupolska D. Clark G. Goch G. Debski J. Floras K. Cantero A.. (2009). The role of annexin 1 in drought stress in Arabidopsis. Plant Physiol. 150, 1394–1410. 10.1104/pp.109.135228 19482919 PMC2705051

[B70] Korver R. A. Koevoets I. T. Testerink C. (2018). Out of shape during stress: a key role for auxin. Trends Plant Sci. 23, 783–793. 10.1016/j.tplants.2018.05.011 29914722 PMC6121082

[B71] Kosma D. Bourdenx B. Bernard A. Parsons E. Lü S. Joubès J.. (2009). The impact of water deficiency on leaf cuticle lipids of Arabidopsis. Plant Physiol. 151, 918–1929. 10.1104/pp.109.141911 19819982 PMC2785987

[B72] Kudo T. Makita N. Kojima M. Tokunaga H. Sakakibara H. (2012). Cytokinin activity of cis-Zeatin and phenotypic alterations induced by overexpression of putative cis-Zeatin-O-glucosyltransferase in rice. Plant Physiol.160, 319–331. 10.1104/pp.112.196733 22811434 PMC3440209

[B73] Laloum T. Martín G. Duque P. (2018). Alternative splicing control of abiotic stress responses. Trends Plant Sci. 23, 140–150. 10.1016/j.tplants.2017.09.019 29074233

[B74] Law C. Chen Y. Shi W. Smyth G. (2014). voom: precision weights unlock linear model analysis tools for RNA-seq read counts. Genome Biol. 15:R29. 10.1186/gb-2014-15-2-r29 24485249 PMC4053721

[B75] Lehti-Shiu M. Zou C. Hanada K. Shiu S. H. (2009). Evolutionary history and stress regulation of plant receptor-like kinase/pelle genes. Plant Physiol. 150, 12–26. 10.1104/pp.108.134353 19321712 PMC2675737

[B76] Lesk C. Rowhani P. Ramankutty N. (2016). Influence of extreme weather disasters on global crop production. Nature 529, 84–87. 10.1038/nature16467 26738594

[B77] Li S. Yu X. Cheng Z. Zeng C. Li W. Zhang L.. (2020). Large-scale analysis of the cassava transcriptome reveals the impact of cold stress on alternative splicing. J. Exp. Bot. 71, 422–434. 10.1093/jxb/erz444 31713628

[B78] Liu Q. Li X. Yan S. Yu T. Yang J. Dong J.. (2018). OsWRKY67 positively regulates blast and bacteria blight resistance by direct activation of PR genes in rice. BMC Plant Biol. 18, 257–270. 10.1186/s12870-018-1479-y 30367631 PMC6204034

[B79] Liu W. J. Yuan S. Zhang N. H. Lei T. Duan H. G. Liang H. G. (2006). Effect of water stress on photosystem 2 in two wheat cultivars. Physiol. Plant. 50, 597–602. 10.1007/s10535-006-0094-1

[B80] Liu Z. Qin J. Tian X. Xu S. Wang Y. Li H.. (2018). Global profiling of alternative splicing landscape responsive to drought, heat and their combination in wheat (*Triticum aestivum* L.). Plant Biotechnol. J. 16, 714–726. 10.1111/pbi.12822 28834352 PMC5814593

[B81] Livak K. Schmittgen T. (2001). Analysis of relative gene expression data using real-time quantitative PCR and the 2–ΔΔCT method. Methods 25, 402–408. 10.1006/meth.2001.1262 11846609

[B82] Lobell D. B. Schlenker W. Costa-Roberts J. (2011). Climate trends and global crop production since 1980. Science 333, 616–620. 10.1126/science.1204531 21551030

[B83] Lombardi M. (2012). The barley expansin family. (Ph.D. dissertation). University of Adelaide, Waite, MN.

[B84] Lopato S. Waigmann E. Barta A. (1996). Characterization of a novel arginine/serine-rich splicing factor in Arabidopsis. Plant Cell 8, 2255–2264. 10.1105/tpc.8.12.2255 8989882 PMC161350

[B85] Lv Y. Xu L. Dossa K. Zhou K. Zhu M. Xie H.. (2019). Identification of putative drought-responsive genes in rice using gene co-expression analysis. Bioinformation 15, 480–489. 10.6026/97320630015480 31485134 PMC6704332

[B86] Marowa P. Ding A. Kong Y. (2016). Expansins: roles in plant growth and potential applications in crop improvement. Plant Cell Rep. 35, 949–965. 10.1007/s00299-016-1948-4 26888755 PMC4833835

[B87] Martin M. (2011). Cutadapt removes adapter sequences from high-throughput sequencing reads. EMBnet J. 17, 10–12. 10.14806/ej.17.1.200

[B88] Mei W. Liu S. Schnable J. C. Yeh C. T. Springer N. M. Schnable P. S.. (2017). A comprehensive analysis of alternative splicing in paleopolyploid maize. Front. Plant Sci. 8:694. 10.3389/fpls.2017.00694 28539927 PMC5423905

[B89] Mejri M. Siddique K. Saif T. Abdelly C. Hessini K. (2016). Comparative effect of drought duration on growth, photosynthesis, water relations, and solute accumulation in wild and cultivated barley species. J. Plant Nutr. Soil Sci. 179, 327–335. 10.1002/jpln.201500547

[B90] Meza I. Siebert S. Döll P. Kusche J. Herbert C. Rezaei E. (2020). Global-scale drought risk assessment for agricultural systems. Nat. Hazards Earth Syst. Sci. 20, 695–712. 10.5194/nhess-20-695-2020

[B91] Mornhinweg D. W. Bregitzer P. P. Porter D. R. Peairs F. B. Baltensperger D. D. Hein G. L. (2009). Registration of Sidney Spring feed barley resistant to Russian wheat aphid. J. Plant Regist. 3, 214–218. 10.3198/jpr2009.04.0205crc

[B92] Mudgil Y. Shiu S. H. Stone S. Salt J. Goring J. (2004). A Large complement of the predicted Arabidopsis ARM repeat proteins are members of the U-Box E3 ubiquitin ligase family. Plant Physiol. 134, 59–66. 10.1104/pp.103.029553 14657406 PMC316287

[B93] Munemasa S. Hauser F. Park J. Waadt R. Brandt B. Schroeder J. (2015). Mechanisms of abscisic acid-mediated control of stomatal Aperture. Curr. Opin. Plant Biol. 28, 154–162. 10.1016/j.pbi.2015.10.010 26599955 PMC4679528

[B94] Nakamura T. Nomura M. Jagendorf A. Ueda A. Takabe T. (2001). An isozyme of betaine aldehyde dehydrogenase in barley. Plant Cell Physiol. 42, 1088–1092. 10.1093/pcp/pce136 11673624

[B95] Nakashima K. Tran L. S. Nguyen D. V. Fujita M. Maruyama K. Todaka D.. (2007). Functional analysis of a NAC-type transcription factor OsNAC6 involved in abiotic and biotic stress-responsive gene expression in rice. Plant J. 51, 617–630. 10.1111/j.1365-313X.2007.03168.x 17587305

[B96] Nakashima K. Yamaguchi-Shinozaki K. Shinozaki K. (2014). The transcriptional regulatory network in the drought response and its crosstalk in abiotic stress responses including drought, cold, and heat. Front. Plant Sci. 5:170. 10.3389/fpls.2014.00170 24904597 PMC4032904

[B97] Narsai R. Wang C. Chen J. Wu J. Shou H. Whelan J.. (2013). Antagonistic, overlapping and distinct responses to biotic stress in rice (*Oryza sativa*) and interactions with abiotic stress. BMC Genomics 14, 93–114. 10.1186/1471-2164-14-93 23398910 PMC3616870

[B98] Nelissen H. Sun X. H. Rymen B. Jukumaru Y. Kojima M. Takebayashi Y. (2018). The reduction in maize leaf growth under mild drought affects the transition between cell division and cell expansion and cannot be restored by elevated gibberellic acid levels. Plant Biotechnol. J. 16, 615–627. 10.1111/pbi.12801 28730636 PMC5787831

[B99] Omena-Garcia R. P. Martins A. O. Medeiros D. B. Vallarino J. G. Ribeiro D. M. Fernie A. R. (2019). Growth and metabolic adjustments in response to gibberellin deficiency in drought stressed tomato plants. Environ. Exp. Bot. 159, 95–107. 10.1016/j.envexpbot.2018.12.011

[B100] Ouyang W. Struik P. Yin X. Yang J. (2017). Stomatal conductance, mesophyll conductance, and transpiration efficiency in relation to leaf anatomy in rice and wheat genotypes under drought. J. Exp. Bot. 68, 5191–5205. 10.1093/jxb/erx314 28992130 PMC5853379

[B101] Ozturk N. Z. Talamè V. Deyholos M. Michalowski C. Galbraith D. Gozukirmizi N.. (2002). Monitoring large-scale changes in transcript abundance in drought- and salt-stressed barley. Plant Mol. Biol. 48, 551–573. 10.1023/A:1014875215580 11999834

[B102] Patro R. Duggal G. Love M. I. Irizarry R. A. Kingsford C. (2017). Salmon provides fast and bias-aware quantification of transcript expression. Nat. Methods. 14, 417–419. 10.1038/nmeth.4197 28263959 PMC5600148

[B103] Pavlu J. Novák J. Koukalová V. Luklová M. Brzobohatý B. Cerný M. (2018). Cytokinin at the crossroads of abiotic stress signalling pathways. Int. J. Mol. Sci. 19, 2450–2486. 10.3390/ijms19082450 30126242 PMC6121657

[B104] Peleg Z. Blumwald E. (2011). Hormone balance and abiotic stress tolerance in crop plants. Curr. Opin. Plant Biol. 14, 290–295. 10.1016/j.pbi.2011.02.001 21377404

[B105] Pieczynski M. Wyrzykowska A. Milanowska K. Boguszewska-Mankowska D. Zagdanska B. Karlowski W. (2018). Genome wide identification of genes involved in the potato response to drought indicates functional evolutionary conservation with Arabidopsis plants. Plant Biotechnol. J. 16, 603–614. 10.1111/pbi.12800 28718511 PMC5787840

[B106] Plaza-Wüthrich S. Blösch R. Rindisbacher A. Cannarozzi G. Tadele Z. (2016). Gibberellin deficiency confers both lodging and drought tolerance in small cereals. Front. Plant Sci. 7:643. 10.3389/fpls.2016.00643 27242844 PMC4865506

[B107] Priya M. Dhanker O. Siddique K. H. M. HanumanthaRao B. Nair R. Pandey S.. (2019). Drought and heat stress-related proteins: an update about their functional relevance in imparting stress tolerance in agricultural crops. Theor. Appl. Genet. 132, 1607–1638. 10.1007/s00122-019-03331-2 30941464

[B108] Quigley F. Rosenberg J. M. Shachar-Hill Y. Bohnert H. (2001). From genome to function: the *Arabidopsis aquaporins*. Genome Biol. 3:research0001. 10.1186/gb-2001-3-1-research0001 11806824 PMC150448

[B109] Ramegowda V. Krishnan A. Pereira A. (2014). Rice GROWTH UNDER DROUGHT KINASE is required for drought tolerance and grain yield under normal and drought stress conditions. Plant Physiol. 166, 1634–1645. 10.1104/pp.114.248203 25209982 PMC4226359

[B110] Rapazote-Flores P. Bayer M. Milne L. Mayer C. D. Fuller J. Guo W.. (2019). BaRTv1.0: an improved barley reference transcript dataset to determine accurate changes in the barley transcriptome using RNA-seq. BMC Genomics 20, 968–985. 10.1186/s12864-019-6243-7 31829136 PMC6907147

[B111] Raudvere U. Kolberg L. Kuzmin I. Arak T. Adler P. Peterson H.. (2019). g: Profiler: a web server for functional enrichment analysis and conversions of gene lists (2019 update). Nucl. Acid. Res. 47, W191–W198. 10.1093/nar/gkz369 31066453 PMC6602461

[B112] Ray S. Dansana P. K. Giri J. Deveshwar P. Arora R.. (2011). Modulation of transcription factor and metabolic pathway genes in response to water-deficit stress in rice. Funct. Integr. Genomic. 11, 157–178. 10.1007/s10142-010-0187-y 20821243

[B113] Reddy A. S. N. Marquez Y. Kalyna M. Barta A. (2013). Complexity of the alternative splicing landscape in plants. Plant Cell 25, 3657–3683. 10.1105/tpc.113.117523 24179125 PMC3877793

[B114] Ritchie M. E. Phipson B. Wu D. Hu Y. Law C. W. Shi W.. (2015). Limma powers differential expression analyses for RNA-sequencing and microarray studies. Nucleic Acids Res. 43:e47. 10.1093/nar/gkv007 25605792 PMC4402510

[B115] Ryu M. Cho S. K. Hong Y. Kim J. Kim J. H. Kim G. M.. (2019). Classification of barley U-box E3 ligases and their expression patterns in response to drought and pathogen stresses. BMC Genomics 20, 326–341. 10.1186/s12864-019-5696-z 31035917 PMC6489225

[B116] Sah S. K. Reddy K. R. Li J. (2016). *Abscisic acid and abiotic stress tolerance in crop* plants. Front. Plant Sci. 7:571. 10.3389/fpls.2016.00571 27200044 PMC4855980

[B117] Sallam A. Alqudah A. Dawood M. Baenziger P. Börner A. (2019). Drought stress tolerance in wheat and barley: advances in physiology, breeding and genetics research. Int. J. Mol. Sci. 20, 3137–3175. 10.3390/ijms20133137 31252573 PMC6651786

[B118] Schäfer M. Brütting C. Meza-Canales I. Großkinsky D. Vankova R. Baldwin I. (2015). The role of cis-zeatin-type cytokinins in plant growth regulation and mediating responses to environmental interactions. J. Exp. Bot. 66, 4873–4884. 10.1093/jxb/erv214 25998904 PMC5147713

[B119] Scharwies J. D. (2017). The role of aquaporins in plant responses to drought. (Ph.D. dissertation). The University of Adelaide, Waite, MN.

[B120] Schmid I. Franzaring J. Müller M. Brohon N. Calvo O. C. Högy P. (2016). Drought stress effects of CO_2_ enrichment and drought on photosynthesis, growth and yield of an old and a modern barley cultivar. J. Agron. Crop Sci. 202, 81–95.

[B121] Schonfeld M. A. Johnson R. C. Carver B. F. Mornhinweg D. W. (1988). Water relations in winter wheat as drought resistance indicator. Crop Sci. 28, 526–531.

[B122] Shaik R. Ramakrishna W. (2012). Bioinformatic analysis of epigenetic and microRNA mediated regulation of drought responsive genes in rice. PLoS ONE 7:e49331. 10.1371/journal.pone.0049331 23145152 PMC3493535

[B123] Sharma M. Pandey G. K. (2016). Expansion and function of repeat domain proteins during stress and development in plants. Front. Plant Sci. 6:1218. 10.3389/fpls.2015.01218 26793205 PMC4707873

[B124] Sharma M. Singh A. Shankar A. Pandey A. Baranwal V. Kapoor S.. (2014). Comprehensive expression analysis of rice Armadillo gene family during abiotic stress and development. DNA Res. 21, 267–283. 10.1093/dnares/dst056 24398598 PMC4060948

[B125] Shim J. S. Oh N. Chung P. J. Kim Y. S. Choi Y. D. Kim J. K. (2018). Overexpression of OsNAC14 improves drought tolerance in rice. Front. Plant Sci. 9:310. 10.3389/fpls.2018.00310 29593766 PMC5855183

[B126] Silveira R. D. D. Abreu F. R. M. Mamidi S. McClean P. E. Vianello R. P. Lanna A. C.. (2015). Expression of drought tolerance genes in tropical upland rice cultivars (*Oryza sativa*). Genet. Mol. Res. 14, 8181–8200. 10.4238/2015.July.27.6 26345744

[B127] Talamé V. Ozturk N. Z. Bohnert H. Tuberosa R. (2007). Barley transcript profiles under dehydration shock and drought stress treatments: a comparative analysis. J. Exp. Bot. 58, 229–240. 10.1093/jxb/erl163 17110587

[B128] Törönen P. Medlar A. Holm L. (2018). PANNZER2: a rapid functional annotation web server. Nucl. Acid Res. 46, W84–W88. 10.1093/nar/gky350 29741643 PMC6031051

[B129] Ullah A. Manghwar H. Shaban M. Khan A. Akbar A. Ali U.. (2018). Phytohormones enhanced drought tolerance in plants: a coping strategy. Environ. Sci. Pollut. Res. 25, 33103–33118. 10.1007/s11356-018-3364-5 30284160

[B130] Vishwakarma K. Upadhyay N. Kumar N. Yadav G. Singh J. Mishra R. K. (2017). Abscisic acid signaling and abiotic stress tolerance in plants: a review on current knowledge and prospects. Front. Plant Sci. 8, 161–173. 10.3389/fpls.2017.00161 28265276 PMC5316533

[B131] Wang X. Chen Z. H. Yang C. Zhang X. Jin G. Chen G.. (2018). Genomic adaptation to drought in wild barley is driven by edaphic natural selection at the Tabigha evolution slope. Proc. Natl. Acad. Sci. U.S.A. 115, 5223–5228. 10.1073/pnas.1721749115 29712833 PMC5960308

[B132] Wang Z. Dane F. (2013). NAC (NAM/ATAF/CUC) transcription factors in different stresses and their signaling pathway. Acta Physiol. Plant 35, 1397–1408. 10.1007/s11738-012-1195-4

[B133] Wu H. Shi S. Lu X. Li T. Wang J. Liu T.. (2019). Expression analysis and functional characterization of CER1 family genes involved in very-long-chain alkanes biosynthesis in *Brachypodium distachyon*. Front. Plant. Sci. 10:1389. 10.3389/fpls.2019.01389 31737015 PMC6838206

[B134] Wu X. L. Shiroto Y. Kishitani S. Ito Y. Toriyama K. (2009). Enhanced heat and drought tolerance in transgenic rice seedlings overexpressing *OsWRKY11* under the control of HSP101 promoter. Plant Cell Rep. 28, 21–30. 10.1007/s00299-008-0614-x 18818929

[B135] Wu Y. Thorne E. T. Sharp R. E. Cosgrove D. (2001). Modification of expansin transcript levels in the maize primary root at lower water potentials. Plant Physiol. 126, 1471–1479. 10.1104/pp.126.4.1471 11500546 PMC117147

[B136] Xiang J. Zhou X. Zhang X. Liu A. Xiang Y. Yan M.. (2018). The Arabidopsis AtUNC-93 acts as a positive regulator of abiotic stress tolerance and plant growth via modulation of ABA signaling and K(+) homeostasis. Front. Plant Sci. 9, 718–733. 10.3389/fpls.2018.00718 29899751 PMC5989354

[B137] Xing H. T. Guo P. Xia X. L. Yin W. L. (2011). PdERECTA, a leucine-rich repeat receptor-like kinase of poplar, confers enhanced water use efficiency in Arabidopsis. Planta 234, 229–241. 10.1007/s00425-011-1389-9 21399949

[B138] Xu Q. Feng W. J. Peng H. R. Ni Z. F. Sun Q. X. (2014). TaWRKY71, a WRKY transcription factor from wheat, enhances tolerance to abiotic stress in transgenic *Arabidopsis thaliana*. Cereal Res. Commun. 42, 47–57. 10.1556/CRC.2013.0051

[B139] Yamaguchi S. (2008). Gibberellin metabolism and its regulation. Annu. Rev. Plant Biol. 59, 225–251. 10.1146/annurev.arplant.59.032607.092804 18173378

[B140] Yan Q. Xia X. Sun Z. Fang Y. (2017). Depletion of Arabidopsis SC35 and SC35-like serine/arginine-rich proteins affects the transcription and splicing of a subset of genes. PLoS Genet. 13:e1006663. 10.1371/journal.pgen.1006663 28273088 PMC5362245

[B141] Yang J. Zhang G. An J. Li Q. Chen Y. Zhao X. (2020). Expansin gene TaEXPA2 positively regulates drought tolerance in transgenic wheat (*Triticum aestivum* L.). Plant Sci. 298, 10.1016/j.plantsci.2020.110596 32771153

[B142] Yang Y. W. Chen H. C. Jen W. F. Liu L. Y. Chang M. C. (2015). Comparative transcriptome analysis of shoots and roots of TNG67 and TCN1 rice seedlings under cold stress and following subsequent recovery: insights into metabolic pathways, phytohormones, and transcription factors. PLoS ONE 10:e0131391. 10.1371/journal.pone.0131391 26133169 PMC4489882

[B143] Zadoks C. J. Chang T. T. Konzak C. F. (1974). A decimal code for the growth stages of cereals. Weed Res. 14, 415–421. 10.1111/j.1365-3180.1974.tb01084.x

[B144] Zeng X. Bai L. Wei,1 Z. Yuan H. Wang Y. Xu Q.. (2016). Transcriptome analysis revealed the drought-responsive genes in Tibetan hulless barley. BMC Genomics 17, 386–398. 10.1186/s12864-016-2685-3 27207260 PMC4875595

[B145] Zhang F. Zhou Y. Zhang M. Luo X. Xie J. (2017). Effects of drought stress on global gene expression profile in leaf and root samples of Dongxiang wild rice (*Oryza rufipogon*). Biosci. Rep. 37:BSR20160509. 10.1042/BSR20160509 28424372 PMC6434088

[B146] Zhang Y. Wang L. (2005). The WRKY transcription factor superfamily: its origin in eukaryotes and expansion in plants. BMC Evol. Biol. 5:1. 15629062 10.1186/1471-2148-5-1 PMC544883

[B147] Zheng Y. Jiao C. Sun H. Rosli H. G. Pombo M. A. Zhang P.. (2016). iTAK: a program for genome-wide prediction and classification of plant transcription factors, transcriptional regulators, and protein kinases. Mol. Plant 9, 1667–1670. 10.1016/j.molp.2016.09.014 27717919

[B148] Zhou J. Wang X. Jiao Y. Qin Y. Liu X.. (2007). Global genome expression analysis of rice in response to drought and high-salinity stresses in shoot, flag leaf, and panicle. Plant Mol. Biol. 63, 591–608. 10.1007/s11103-006-9111-1 17225073 PMC1805039

[B149] Zwack P. Rashotte A. (2015). Interactions between cytokinin signalling and abiotic stress responses. J. Exp. Bot. 66, 4863–4871. 10.1093/jxb/erv172 25911740

