# Supplementary material for: The Effect of Drought on Transcriptome and Hormonal Profiles in Barley Genotypes With Contrasting Drought Tolerance
Source: Front Plant Sci. 2020 Dec 23;11:618491. doi: 10.3389/fpls.2020.618491 (PMC7786106; doi:10.3389/fpls.2020.618491)
Supplement: Supplementary file 1 [file Supplementary_Figure_1.docx]

**
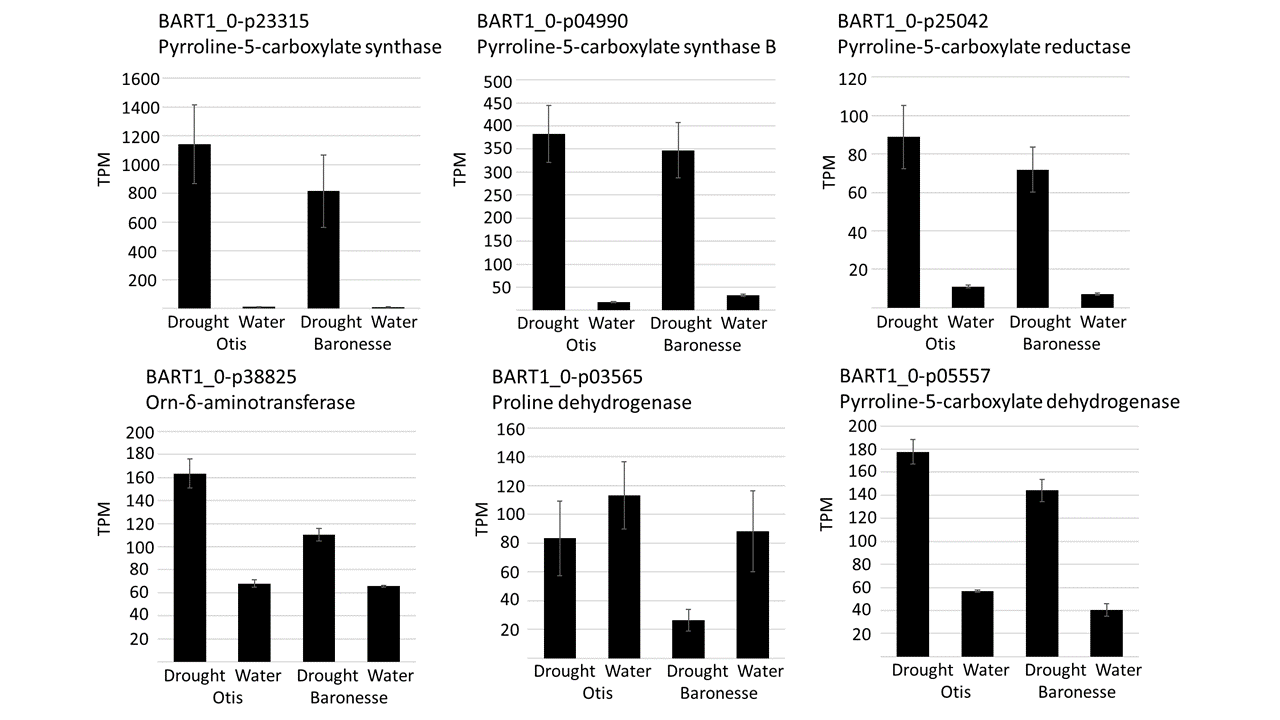
**

**Supplementary Figure 1.** Differential expression of proline biosynthesis and turnover genes between the two genotypes Otis and Baronesse at the initial wilting stage compared to well-watered conditions. Proline is produced by reduction of glutamate, catalyzed by pyrroline-5-carboxylate synthase (Barley orthologues, BaRT1_0-p23315; HORVU3Hr1G085760 and BaRT1_0-p04990; HORVU1Hr1G072780) and pyrroline-5-carboxylate reductase (barley orthologue, BaRT1_0-p25042; HORVU3Hr1G106720) or from ornithine, catalyzed by Orn-δ-aminotransferase (barley orthologue, BaRT1_0-p38825; HORVU5Hr1G092960). Proline content is further regulated by turnover through proline dehydrogenase (barley orthologue, BaRT1_0-p03565; HORVU1Hr1G053440) and pyrroline-5-carboxylate dehydrogenase (barley orthologue, BaRT1_0-p05557; HORVU1Hr1G080320) (Verbruggen & Hermans, 2008). Differential gene expression analysis showed large scale changes in gene expression in both genotypes consistent with a significant increase in proline in both genotypes at the initial wilting stage.
