# Supplementary figures and images for: The Effect of Drought on Transcriptome and Hormonal Profiles in Barley Genotypes With Contrasting Drought Tolerance
Source: Front Plant Sci. 2020 Dec 23;11:618491. doi: 10.3389/fpls.2020.618491 (PMC7786106; doi:10.3389/fpls.2020.618491)

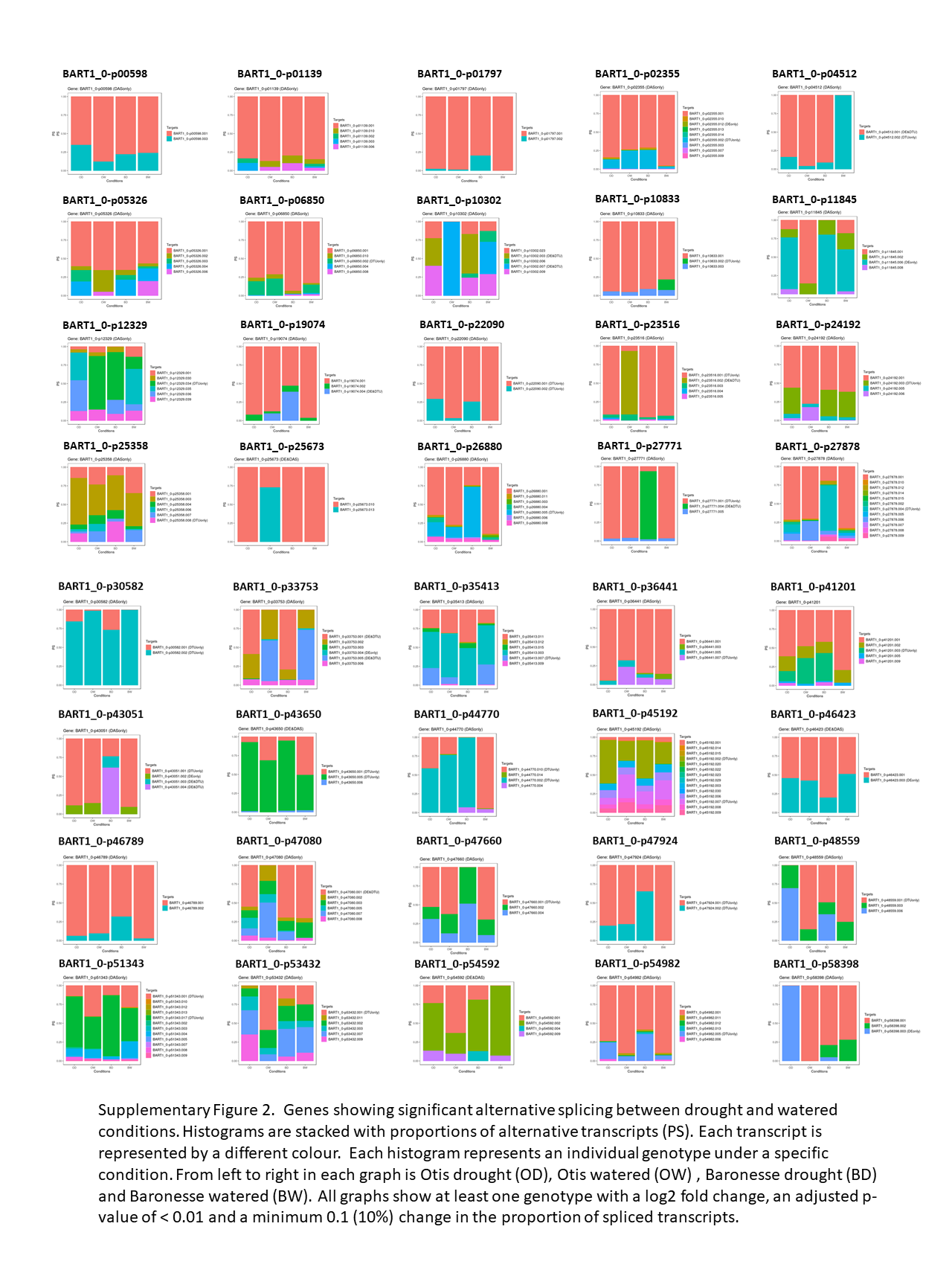

Supplement: Supplementary file 2 [file Image_2.png]
